# The interaction of focus and phrasing with downstep and post-low-bouncing in Mandarin Chinese

**DOI:** 10.3389/fpsyg.2022.884102

**Published:** 2022-09-30

**Authors:** Bei Wang, Frank Kügler, Susanne Genzel

**Affiliations:** ^1^Key Laboratory of Language, Cognition and Computation, School of Foreign Languages, Beijing Institute of Technology, Beijing, China; ^2^Department of Linguistics, Goethe University Frankfurt, Frankfurt, Germany; ^3^i2x GmbH, Berlin, Germany

**Keywords:** downstep, post-low-bouncing, phrasing, focus, intonation, Mandarin Chinese

## Abstract

L(ow) tone in Mandarin Chinese causes both downstep and post-low-bouncing. Downstep refers to the lowering of a H(igh) tone after a L tone, which is usually measured by comparing the H tones in a “H…HLH…H” sentence with a “H…HHH…H” sentence (*cross-comparison*), investigating whether downstep sets a new pitch register for the scaling of subsequent tones. Post-low-bouncing refers to the raising of a H tone after a focused L tone. The current study investigates how downstep and post-low-bouncing interact with focus and phrasing in Mandarin Chinese. In the experiment, we systematically manipulated (a) the tonal environment by embedding two syllables with either LH or HH tone (syllable X and Y) sentence-medially in the same carrier sentences containing only H tones; (b) boundary strength between X and Y by introducing either a syllable boundary or a phonological phrase boundary; and (c) information structure by either placing a contrastive focus in the HL/HH word (XF), syllable Y (YF), or the sentence-final word (ZF). A wide-focus condition served as the baseline. With systematic control of focus and boundary strength around the L tone, the current study shows that the downstep effect in Mandarin is quite robust, lasting for 3–5 H tones after the L tone, but eventually levelling back again to the register reference line of a H tone. The way how focus and phrasing interact with the downstep effect is unexpected. Firstly, sentence-final focus has no anticipatory effect on shortening the downstep effect; instead, it makes the downstep effect lasts longer as compared to the wide focus condition. Secondly, the downstep effect still shows when the H tone after the L tone is on-focus (YF), in a weaker manner than the wide focus condition, and is overridden by the post-focus-compression. Thirdly, the downstep effect gets greater when the boundary after the L tone is stronger, because the L tone is longer and more likely to be creaky. We further analyzed downstep by measuring the F0 drop between the two H tones surrounding the L tone (*sequential-comparison*). Comparing it with F0 drop in all-H sentences (i.e., declination), it showed that the downstep effect was much greater and more robust than declination. However, creaky voice in the L tone was not the direct cause of downstep. At last, when the L tone was under focus (XF), it caused a post-low-bouncing effect, which is weakened by a phonological phrase boundary. Altogether, the results showed that although intonation is largely controlled by informative functions, the physical-articulatory controls are relatively persistent, varying within the pitch range of 2.5 semitones. Downstep and post-low-bouncing in Mandarin Chinese thus seem to be mainly due to physical-articulatory movement on varying pitch, with the gradual tonal F0 change meeting the requirement of smooth transition across syllables, and avoiding confusion in informative F0 control.

## Introduction

Intonation carries communicative functions, such as focus and phrasing, but much of intonation variation also comes from tonal interactions. To better understand the interaction of tone and intonation, it is important to take into account of both informative and articulatory effects (Xu et al., 2012). In Mandarin, for instance, L tone causes *pre-low-raising, post-low-bouncing,* and *downstep* in the surrounding H tones. Pre-low-raising refers to the pitch raising in the H tone preceding the L tone (Lee et al., 2021). Post-low-bouncing is the phenomenon that F0 of the post-low syllables suddenly goes up first, then drops back gradually, in the condition that the following syllables carry neutral tones or the L tone is under focus (Shen, 1994; Chen and Xu, 2006; Gu and Lee, 2009; Prom-on et al., 2012). Downstep refers to the downtrend of F0 caused by L tones, in the way that the H tones after a L tone is with lower F0 than previous H tones (Xu, 1997, 1999; Shih, 2000; Laniran and Clements, 2003; Connell, 2011). The first two tonal effects have been extensively studied and well explained with articulatory movement of pitch control (Prom-on et al., 2012; Lee et al., 2021).

In this paper, our main goal is to study *downstep* in Mandarin, and its interaction with focus and phrasing. To be more specific, we aim to study how on-focus raising and post-focus compression (PFC) in F0 interact with downstep, and if a phrase boundary terminates downstep. Moreover, considering the presence of global F0 declination in all-H sentences (Shih, 2000; Yuan and Liberman, 2014), we investigate whether downstep and declination share the same pitch lowering mechanism. To go further, a L tone in Mandarin is commonly accompanied by creaky voice (Kuang, 2017, 2018), we thus investigate whether creaky voice may cause downstep. Thirdly, we aim to study the interaction of boundary with post-L-bouncing, which happens when a L tone is focused. Our investigation tackles the question whether a phonological phrase boundary cancels post-L-bouncing or not.

The next section starts with a review of downstep and declination followed by a review on post-low-bouncing, and then focus and phrasing. To better understand pitch from both linguistic and articulatory perspectives, we also briefly introduce a review on laryngeal movement of varying pitch. In the end of section “Background,” the research questions are summarized.

## Background

### Downstep and declination

There is a global downtrend or declination in a sentence (e.g., Gussenhoven, 2004). Articulatorily, declination is arguably caused by a decrease in subglottal pressure over time (Lieberman, 1967; Collier, 1975; Pierrehumbert 1980; Gelfer et al., 1983). Beside declination, lexical tones and tonal interactions also cause downtrend in F0 contours, e.g., downstep lowers the following H tones. Downstep has long been discussed in African languages (Yoruba (Niger-Congo): Ward, 1952; *cf.* Courtenay, 1971; Luo (Nilotic): Tucker and Creider, 1975; Twi (Akan): Stewart, 1965; Genzel and Kügler, 2011; Kügler 2017; Tswana (Southern Bantu): Zerbian and Kügler, 2015, 2021; among many others). In the African linguistic tradition, downstep is distinguished from downdrift (Stewart, 1965; Hombert, 1974; see Hyman and Leben, 2017 for an overview), terrace (Courtenay, 1971), or automatic and non-automatic downstep (see detailed discussion in Connell, 2001; Rialland and Somé, 2011; Leben, 2014). Strictly speaking, downstep refers to a new register or ceiling established for subsequent H tones after a L tone (Snider, 1990; Snider and van der Hulst, 1993; Connell, 2017; *cf.* Akumbu, 2019). The differentiation between downstep and downdrift, or automatic and non-automatic downstep concerns the fact that in several African languages, both an overtly realized L tone and a floating L tone functions as the trigger of the lowering process. A floating L tone triggers non-automatic downstep, whereas a phonetically realized L tone triggers automatic downstep (Hyman and Leben, 2017). Phonetically, no difference is found between these two types of downstep (e.g., Genzel and Kügler, 2011; Kügler 2017 for Akan). Since there is no floating L tone in Mandarin Chinese, we do not need to differentiate them. We here take the broad definition of downstep as the lowering of F0 after a L tone, following Shih (1988); Xu (1999); Laniran and Clements (2003), and Genzel (2013) among many others, see (1).

1. Downstep: In a HLH tone sequence, the second H is realized with lowered F0 compared to the first H, due to the L tone (*sequential-comparison*). The size of downstep can be paradigmatically calculated as the difference of F0-maximum in the H tones after a L tone and in the corresponding H tones of an all-H tone phrase (*cross-comparison*).

In some West-African languages, downstep initiates a new pitch register to which subsequent tones are scaled, phonologically termed as register tones (e.g., Snider, 1998) or register features (e.g., Akumbu, 2019). In Mandarin, there is no study directly concerning the effect of downstep as setting up a new register tone or register line. We here introduce three studies, which suggest that downstep in Mandarin does not seem to set up a new register tone. First, it showed that several H tones after a L are lowered in F0 as compared to all H-tone sentences, then the pitch gradually reaches the target in the all-H sentence toward the end of the sentence (see Figure 4, pp. 66 in Xu, 1999). Second, Gu and Lee (2009) found that the lowering effect in the H tones is greater when the preceding L tone is lower. Third, Wang and Xu (2011) used sentence with HLHL…HL and LHLH…LH tone sequences, the sentence-medial H tones reach roughly the same height as the corresponding H tones in an all-H sentence, which is explained as the balance between pre-Low raising and downstep. Thus, downstep seems to be a tonal feature with gradual change in pitch. A terracing pattern of H tones in the LH sequence—as found in the West-African pattern—does not seem to exist in Mandarin Chinese.

What lacks in previous studies is that how downstep interacts with other informative functions, e.g., prosodic boundary and focus. The first question relates to the domain of downstep. The domain of downstp appears to vary across languages. In Kishamba, morpheme boundaries act as a trigger of downstep (Odden, 1986). In Tswana (Southern Bantu), downstep occurs between prosodic words within a phonological phrase, whereas phonological phrase boundaries block downstep (Zerbian and Kügler, 2015, 2021). In Yoruba, downstep applies across all boundaries within a breath group, which could roughly be interpreted as an intonation phrase (Courtenay, 1971). In Japanese, only an accented word (H*L) within a Major Phrase (MaP) triggers downstep (Pierrehumbert and Beckman, 1988; Selkirk and Tateishi, 1991). The downstep effect in Mandarin as reported in Xu (1999) showed that a phrase boundary does not seem to block downstep, though no systematic data on this issue was provided.

As for the interaction of downstep and focus, we here introduce two studies. Ishihara (2007) studied downstep systematically with sentences in the structure as N1 + N2 + N3 + VP (N and VP are abbreviations of noun and verb phrase respectively). It showed that downstep between N2 and N3 is only partially reset, when N3 is focused and when the syntactic boundary is stronger between N2 and N3. It indicated that downstep is weakened by a strong phrase boundary, and a focused H tone after the L tone. Xu (1999) has shown similar results in Mandarin that the size of downstep seems to be reduced when the H tone after the L tone is focused.

It has been also found that downstep can be canceled in yes/no questions in Hausa (Lindau, 1986), meaning that final F0 raising may counter-balance the downstep effect. In Mandarin, however, it does not seem to be the case as shown in Xu (1999). It requires more systematic analysis on whether sentence final F0 raising interferes with the downstep effect.

As mentioned above, another term easy to be confused with downstep is declination, which refers to the F0 downtrend from the beginning through the end of an utterance. We can see that the crucial difference between declination and downstep is its scope. While declination is a gradual lowering of F0 within an intonation phrase, downstep is a local lowering of F0. Declination has been found in both non-tonal languages (‘t Hart & Cohen, 1973; Maeda, 1976; Cooper and Sorensen, 1977; Pierrehumbert, 1979; Sorensen and Cooper, 1980; Cohen et al., 1982; Umeda, 1982; Ladd 1988) and tonal languages (Cantonese: Zhang, 2017; Ge and Li, 2018; Chinese: Xu, 1999; Shih, 2000; Shih and Lu, 2010). Some researchers argue that declination is a fundamental effect in human speech due to a drop in subglottal air pressure (Lieberman, 1967; Collier, 1975; Pierrehumbert, 1979; Gelfer et al., 1983; Gussenhoven, 2004). However, other researchers stated that declination is a combined effect from different functions, e.g., sentence stress and terminal fall (Lieberman and Tseng, 1980; Xu, 1999; Liu and Xu, 2005), topic initial F0 raising (Umeda, 1982; Wang and Xu, 2011) and discourse structure (Hirschberg and Pierrehumbert, 1986; Nakajima and Allen, 1993; Sluijter and Terken, 1993). Downstep and pre-low bouncing, as introduced earlier, also contribute to the overall declination (Liberman and Pierrehumbert, 1984; Pierrehumbert and Beckman, 1988; Shih, 1988; Xu, 1999). Shih (2000) used sentences with the tone sequence of LRH…HN (L, R, H and N stands for low, rising, high and neutral tone respectively), and found that the H tones show declination in the way that the lowering slope is steeper in shorter sentences, after taking apart focus and final lowering. In Shih and Lu (2010) the intonation of an all H tone digital string (338–811-3783) drops from 300 Hz to almost 100 Hz. Similarly, Yuan and Liberman (2014) found that shorter utterances have steeper declination in both the top line and the baseline, after excluding the initial rising and final lowering effects. They are in favor of the idea that declination is linguistically controlled, but not just a by-product of the physics and physiology of talking. It is possible that the declination in the previous three studies still involves some other unknown effects which are hidden by the regression model. In the current study, we calculated declination syllable-by-syllable, as the F0 drop between two adjacent H tones.

### Post-low-bouncing

A L tone could also cause F0 raising after it, especially when the following syllables carry the neutral tone, termed as post-low-bouncing (Mandarin and Cantonese: Chao, 1968; Lin and Yan, 1980; Shih, 1988; Chen and Xu, 2006; Gu and Lee, 2009; *cf.* Prom-on et al., 2012). As discussed in Prom-on et al. (2012), post-low-bouncing has been considered mostly as an articulatory phenomenon, limited to the first neutral tone after the low tone. They emphasized that post-low F0 bouncing is different from a carryover effect, although it occurs between tones. The carryover effect shows in the way that the initial F0 of a syllable is heavily assimilated to the final F0 of the preceding tone, but over the course of the current syllable, F0 gradually approaches its own tonal target. To account for such assimilatory effect, Xu and Wang (2001) proposed the Target Approximation model, which represents the production of successive tones as a process of asymptotically approaching each tonal target within the time interval of the respective syllable, starting from the offset F0 of the preceding syllable. Post-low-bouncing, instead, is the process that pitch increases first then drops back to the underlying target. They discussed the possible physical mechanism behind the low-bouncing effect and suggested a *balance-perturbation hypothesis*. In simple words, after producing a very low F0, the extrinsic laryngeal muscles, especially the sternohyoids (Ohala, 1972; Atkinson, 1978), stop contracting and thus temporarily tip the balance between the two antagonistic forces maintained by the intrinsic laryngeal muscles, resulting in a sudden increase of the vocal fold tension (Prom-on et al., 2012, pp. 422). It still requires articulatory studies to verify the *balance-perturbation hypothesis*. From pitch analysis, one way to test it is to vary syllable duration in the L tone. A longer L tone may reduce post-low-bouncing as it gives more time for the muscles releasing the force. We hence predict that post-low-bouncing is weakened if the L tone is at a phrase boundary, as the L tone is with final lengthening.

### Focus and phrasing

There has been extensive research on how focus is realized prosodically in many languages (for an overview see Kügler and Calhoun, 2020). In Mandarin, focus is realized by increasing the pitch range, intensity, duration and articulatory fullness of the focused word, and reducing the F_0_ and intensity of the following words (post-focus-compression, PFC), while leaving the pre-focus words largely unchanged (Xu, 1999; Chen and Gussenhoven, 2008; Wang and Xu, 2011). Although Mandarin is tonal, its prosodic focus pattern is very similar to English (Cooper et al., 1985; de Jong, 1995; Xu and Xu, 2005), German (Féry and Kügler, 2008) and many other Indo-European languages (Xu et al., 2012). A recent study found that PFC can go across a relative strong prosodic boundary in Mandarin (e.g., a boundary between to clauses), indicating that phrasing does not interfere with post-focus constituents (Wang et al., 2018b). In other words, focus and phrasing are largely encoded in parallel in intonation, though focus may cause prosodic boundaries in some languages (e.g., Kügler and Calhoun, 2020).

Prosodic boundaries are generally indicated by different phonetic cues such as pre-boundary lengthening, silent pause, F0 reset, phonological boundary tones and changes in voice quality (for detailed discussion, see Wang et al., 2018b). In Mandarin Chinese, boundary strength is realized with gradient means rather than categorical ones, differentiated mainly in pre-boundary lengthening and optional silent pause, but not F0 (Xu and Wang, 2009; Wang et al., 2018b). Although pitch reset has been found at a strong boundary (Dutch: de Pijper and Sandeman, 1994; Swerts, 1997; English: Ladd, 1988), F0 plays a limited role to distinguish boundary strength in Mandarin Chinese when tones and focus are carefully controlled (Xu and Wang, 2009; Wang et al., 2018b). Minimum F0 is lowered at a strong boundary with a silent pause for about 200 ms, but not at a phrase boundary within a sentence (Wang et al., 2018b).

It has been found in many languages that pre-boundary syllables are longer than non-final syllables (e.g., English: Byrd, 2000; Finnish: Nakai et al., 2009; Dutch: Swerts and Geluykens, 1994), and articulatory gestures have slower velocity (Krivokapic and Byrd 2012). The prolonged syllable might give rise to fully realized phonetic targets (Lindblom, 1990; DiCanio et al., 2021), e.g., tones in Mandarin (see Figure 4 in Wang et al., 2018b, p. 36). Phrase-final tones carry both lexical tone and post-lexical tone, e.g., a pitch accent and a boundary tone (Arvaniti and Fletcher, 2020). On the other hand, phrase-final position may also be the locus of glottalization (Huffman, 2005), devoicing (Wagner, 2002), and a gradual decay in intensity and F0 (Gussenhoven, 2004; Ladd, 2008; *cf.* DiCanio et al., 2021).

Relating to the current study, we aim to find out whether a phonological phrase boundary reduces or even blocks downstep and post-low-bouncing effect, assuming that the pre-boundary syllable carrying a L tone is longer and hence the tone is fully realized with pitch raising toward the end of the syllable, since fall-rise is the citation form of the L tone in Mandarin. Another possibility is that pitch goes lower when the L tone is longer, thus makes a greater downstep effect.

### Laryngeal movement of varying pitch

After introducing the studies on the linguistic meaning of tone and intonation, we here would like to go back to articulatory studies on pitch control. It will help us to understand downstep and post-low-bouncing, since down to the bottom of the questions raised above, it is all about how the muscles, bones, vocal folds and brain cooperate to realize the pitch targets. The observed pitch contours reflect both linguistic meanings and articulatory constrains.

Yuan and Liberman (2014) discussed articulatory studies on how F0 is controlled. We here just briefly cite some most relevant studies. F0 is determined by the stiffness and effective mass of the vocal folds and the subglottal air pressure (Murry, 1971; Hollien, 1974, 1983; Baer, 1979; Titze, 1988; Stevens, 2000; Zhang, 2016). Intrinsic laryngeal muscles, especially the cricothyroid muscle (CT), are the main contributor to the adjustment of the stiffness and effective mass of the vocal folds. The contraction of CT raises F0; the relaxation of CT, along with the activity of other laryngeal muscles, lowers F0 (Collier, 1975; Atkinson, 1978). Extrinsic laryngeal muscles, which suspend and support the larynx, can also change the states of the vocal folds through vertical larynx movement (Ohala 1972; Honda 1995; Hirose 1997), and F0 falls as the larynx moves down.

F0 lowering is not only accompanied by larynx lowering, relating to extrinsic laryngeal muscles (Honda, 1995; Hirose, 1997) but also involves the joint supraglottal action (Lindqvist-Gauffin, 1969, 1972; *cf.* Lindblom, 2009). In Mandarin, the basic role of larynx height in the execution of tone is complicated by the relationship of larynx height to the state of the larynx: constriction of the supra-glottal laryngeal structures is facilitated by raising the larynx (Edmondson and Esling, 2006) and inhibited by lowering the larynx (Moisik et al., 2014; Moisik and Esling, 2014). Moisik et al. (2014) shows that a L tone target can be reached either by lowering the larynx, or by combining the raise of larynx height and laryngeal constriction, which may lead to creakiness in the low tone. They show that producing the H tone requires any tone involving lowering in pitch is easily becoming creaky, especially the L tone (Kuang, 2017, 2018).

Ladefoged (1973, p. 75) suggested that the creaky voice phonation mechanism is that “because the arytenoid cartilages move forward as they come together; the vocal cords tend to be less stretched in creaky voiced sounds; they are therefore likely to vibrate at a lower frequency. But the coming together of the arytenoids and the movements of the thyroid cartilage that stretch the vocal cords are independent laryngeal gestures, so that it is quite possible for creaky voiced sounds to occur on any pitch.” Creaky voice in Mandarin L tone exhibits various laryngealization properties in acoustic waveforms, including aperiodicity, period doubling, or low-frequency pulse-like vibratory patterns (Gerratt and Kreiman, 2001; Keating et al., 2015). In Mandarin, creaky voice relates to the low target in pitch that the L tones are less creaky when the pitch range is raised, but creakier when the pitch range is lowered (Kuang, 2017, 2018). In previous studies on downstep and post-low-bouncing, creaky voice is usually not taken into account. A consequence of this discussion leads to the question whether creakiness causes downstep or not.

### Research questions and hypotheses

The main goal of the current study is to understand the property of downstep in Mandarin. The second goal is to provide some analysis on how post-low-bouncing interacts with boundary strength. These will lead us to better understand how intonation is shaped by both informative functions and articulatory constrains. The research questions and hypotheses are summarized as the following.

1. How do focus and boundary interact with downstep? We divide this question into 6 sub-questions.
  Q1: Does downstep set up a new register tone?
  According to Xu (1999), we predict that downstep effect lasts for several syllables and approach the all-H reference line gradually in wide focus condition.
  Q2: Does a sentence-final focus terminates downstep?
  We predict that the answer is no because downstep is presumably local, and pitch target is realized syllable-by-syllable as stated in PENTA model (Xu et al., 2022).
  Q3: Is downstep eliminated by on-focus F0 raising and post-focus-compression?
  We predict that informative functions of intonation may override an articulatory effect.
  Q4: How does a phonological phrase boundary interact with downstep?
  Given that pre-boundary L is lengthened, the tonal target is expected to be fully realized, and in turn, that may lead to greater downstep effect, since the L tone is lower or even being creaky.
  Q5: Do declination and downstep share the same mechanism?
  The answer to this question actually depends on how to measure declination and downstep. It also remains controversial whether there is any separate articulatory mechanism controlling declination. Our prediction is that downstep and declination may come from different articulatory control, since downstep is local whereas declination is global.
  Q6: Is creaky voice the cause of downstep?
  Downstep is caused by a L tone, which is usually creaky in Mandarin (Kuang, 2017). It is possible that creaky voice is the main cause of downstep.
2. When a L tone is under focus, post-low-bouncing is expected. Does a phrase boundary block post-low-bouncing (Q7)?
  According to *balance-perturbation hypothesis* (Prom-on et al., 2012), we predict that post-low-bouncing is weakened if the L tone is at a phrase boundary.

## Materials and methods

The experiment aimed to study the size and scope of downstep and post-low-bouncing in Mandarin Chinese, concerning its interaction with focus and phrasing. The size of downstep and post-low-bouncing effects was measured by comparing sentences with all H tones and a comparable sentence with a L tone inserted at the target position, while keeping the rest of the two sentences exactly the same. In this way, we can test whether downstep sets up a new pitch register, as taken the all-H sentence for reference. We named it as *cross-comparison* to answer Q1-Q4. Besides, we also calculated the F0 difference between the two H tones surrounds the L tone, and compared it with the F0 lowering in all-H sentences. We named it as *sequential-comparison* to answer Q5. Thus, the property of downstep and declination can be compared. Moreover, downstep effect caused by creaky and normal L tones were compared, to answer Q6. Post-low-bouncing only occured in the condition of the L tone being focused, thus focus condition is fixed. Only the boundary after the L tone was varied to test whether a strong boundary ends post-low-bouncing (Q7).

### Reading materials

The carrier sentences contained only H tones, except for a neutral tone at sentence-final position. Two target words were embedded in the middle of the carrier sentence, one consisted of a LH word (named as syllable X and Y) triggering downstep and post-low-bouncing, and the other one consisted of a HH word, serving as the reference. The two sentences of each item were read in varied contexts eliciting 4 different focus, and 2 boundary conditions.

Three variables were independently manipulated in this experiment, that is, tone of syllable X (either H or L tone), boundary strength between syllable X and Y (syllable boundary or phrase boundary) and focus type (wide focus (WF), focus on syllable X (XF), on syllable Y (YF) and in sentence final position (ZF)). One set of the sentences in the condition of syllable and phrase boundary were provided in (1a) and (1b). Each sentence was with the syntactic structure as S-V1-O1-V2-O2, and the target words (syllables X and Y) were put in the O1 and V2 position, respectively. Here, by comparing the F0 of syllable Y and that of the following H tones between the two sentences (LH and HH), we can calculate the effect size and scope of downstep. The statistical analysis will then test for how many syllables after the L tone the downstep effect lasts, with consideration of boundary and focus conditions. 
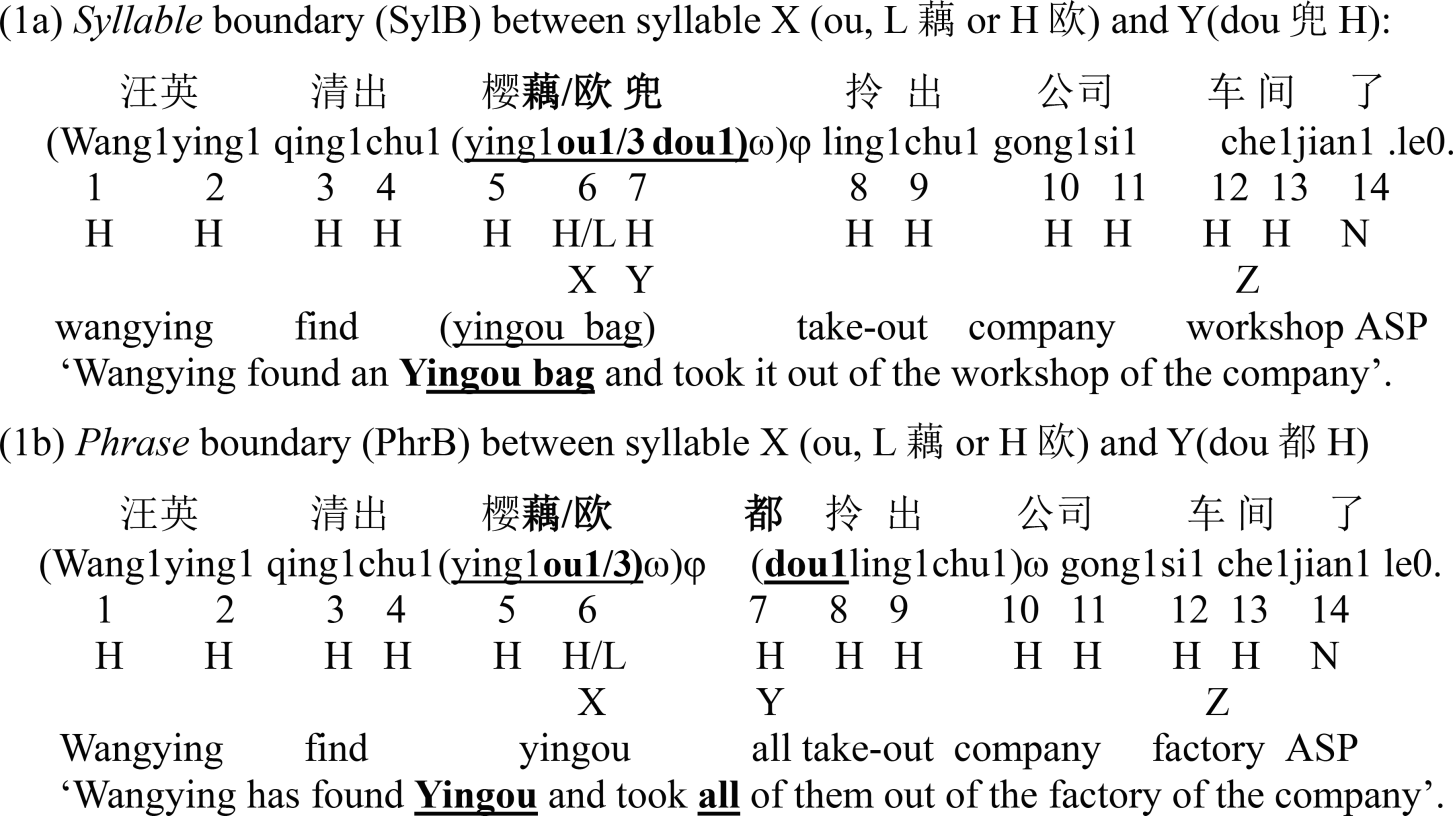


For the two boundary conditions, a monosyllabic homophone of the target syllable Y was used to construct sentences with different syntactic boundaries. Based on the assumption of the syntax-phonology interface, prosodic boundaries, in particular in this experimental setting, are the result of matching syntactic constituents onto prosodic constituents (Selkirk, 2011). Thus, in the syllable boundary condition (1a), the HL_X_H_Y_ was one word, whereas in the phrase boundary condition (1b), the HL_X_ was a word, and the following H_Y_ was an adverb, phrased together with the following words as a verb phrase (VP). Thus, prosodic boundary in condition (1a) was weaker than that in (1b), named as a syllable boundary (SylB) and a phrase boundary (PhrB) respectively. In example (1a), the HHH sequence (yin1ou1dou1樱欧兜, Ying1ou1 bag1) meant a bag printed with ying1ou1 (a make-up word for an exotic plant), whereas in (1b), ‘dou1’ in the HHH sequence (ying1ou1.dou1樱欧都, Ying1ou1 all1) was an adverb, meant “all” to modify the following verb “lingchu (take-out).” In this way, the two boundary conditions were clearly distinguished by using two different characters (兜 vs. 都, bag vs. all). It was the same construction for the HLH sequence, in which the HL tone word is ying1ou3 (樱藕Ying1ou3), which was also a make-up word for an exotic plant. Here, the contrast of syllable X, either being L or H toned, was straightforward by using the two different characters (藕vs 欧, ou3 vs. ou1). In this way, no specific explanation of the material was necessary for the speakers. They were easily able to read the sentences with different tones and phrasing conditions in a natural way.

Focus was elicited by varying a preceding background sentence, which required a correction of the corresponding word in the target sentence. Taken the HL_X_H_Y_ sentence in the syllable boundary condition (see 1a), the four focus conditions are presented in (2). Here, the H tone (syllable Y) is critical to test the effects of downstep and post-low-bouncing, and their interaction with focus. Thus, syllable Y was manipulated as either post-focus (focus on syllable X), on-focus (focus on syllable Y) or pre-focus (focus on syllable Z). A wide focus condition served as the baseline. Similar contexts were constructed for the other sentences, see Appendix I for the whole sentence sets.

The background sentences of the four focus conditions for the sentence (1a) are as follows.

Wide focus: “ni3 ting1shuo1 le0 ma0?” (Have you heard about it?)
X-focus: “bu2shi4ying1an1” (It is not “Yingan.”)
Y-focus: “bu2shi4bao1” (It is not the tote.)
Z-focus: “bu2shi4lou2dao4” (It is not the corridor.)

We constructed two sets of items. In total, 2 (tone of syllable X) × 2 (boundary between X and Y) × 4 (focus) × 2 (sets) × 3 (repetitions) × 8 (speaker) = 768 sentences were analyzed.

### Speakers

Eight native Mandarin speakers participated in the experiment at Minzu University of China (5 female and 3 male speakers), from the age of 20 to 28. They were born and brought up in Beijing, spoke no other Chinese dialects and reported no hearing or speaking impairments. They were paid with small amount of money for taking part in the experiment.

### Recording procedure

The subjects were recorded individually in the speech lab at Minzu University of China. They were asked to read aloud both the context and the target sentences at a normal speed and in a natural way. They sat before a computer monitor, on which the test sentences were displayed, using AudiRec, a custom-written recording program. To make the reading task a little easier for the speakers, the focused words were highlighted with color. A Shure 58 Microphone was placed about 10 cm in front of the speaker. All sentences were digitized directly into a Thinkpad computer and saved as WAV files. The sampling rate was 48 KHz and the sampling format was one channel 6-bit linear. Each speaker repeated the whole set of sentences 3 times in different random order, with about 5 minutes break between sessions. Before the formal recording, they read the sentences silently to get familiar with them, and to make sure that they understood the meaning. The total recording time was about an hour.

### Acoustic measurements and statistical methods

The target sentences were extracted and saved as separate WAV files. ProsodyPro (Xu, 2013) running under Praat (Boersma and Weenink, 2013–2022), was used to take F0 and duration of each syllable measurements from the target sentences, which were all segmented into syllables manually, and at the same time hand-checked vocal cycles markings generated for errors, such as double-marking and period skipping. ProsodyPro then generated syllable-by-syllable F0 contours that were either time-normalized or in the original time scale. At the same time, the script extracted various measurements, including maximum F0, minimum F0 and duration of each syllable. We could measure F0 at the offset of a syllable, however maximum F0 is toward the very end of the syllable (see Figure 5), it is highly probable that the two values are with very little difference. Maximum F0 is much more widely applied in previous studies (e.g., Xu, 1999; Genzel and Kügler, 2011; Prom-on et al., 2012). Thus, we choose maximum F0 to measure downstep effect.

The statistic tests were carried out in the R environment (R Core Team, 2016) by using lme4 package Version 1.1–18 (Bates, et al., 2016) to estimate the effect of the fixed factors (tone, boundary and focus) and the random factors (speaker and sentence set) on the acoustic parameters, e.g., maximum F0 and duration. Regression coefficients (bs), standard errors (SEs) and *t*-values (*t* = b/SE) are reported, taken t > 2.0 as reaching the significant level at *p* < 0.05 (Gelman and Hill, 2006). In the results, we reported the best-fit model according to the model comparisons with the lowest AIC and BIC. For the fixed factors, we took the model with interaction only when there was significant interaction.

**Figure 1:**
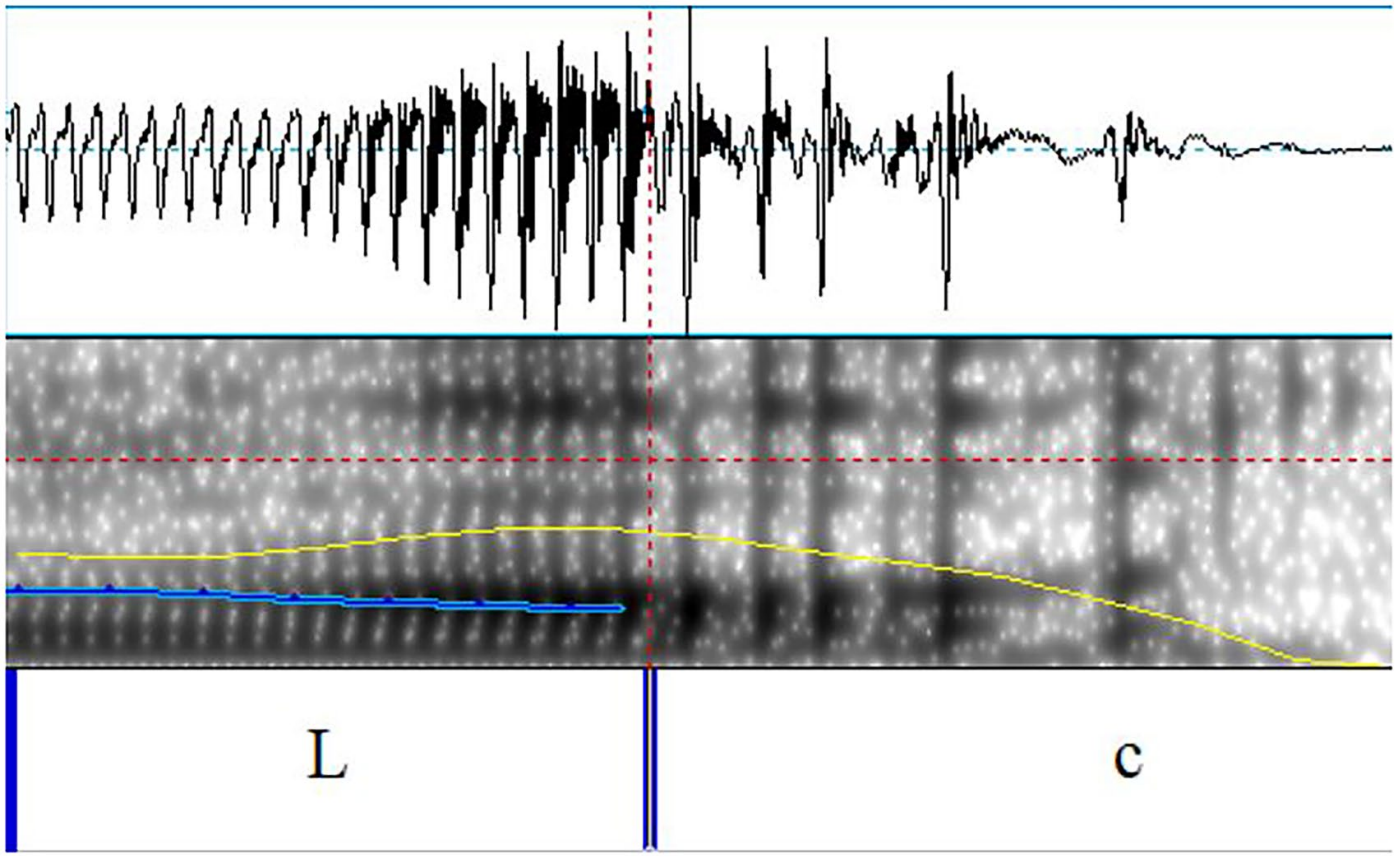
An example of the syllable with creaky L tone. Here, L and c stand for the part with periodic and aperiodic pulses.

Creaky L tone was visually identified by checking the spectrum and the WAV files. Zhang (2016) distinguished four types of creaky voice (see Figure 3 in that paper). We grouped all these types as creaky voice. Since F0 is the main concern in this paper, we here labeled the part with aperiodic pulses as creaky, see Figure 1. In this way, the part of the regular pulses was used to get the F0 values of the syllable. Most of the creaky L tone was similar to what Figure 1 shows.

## Results

**Figure 2:**
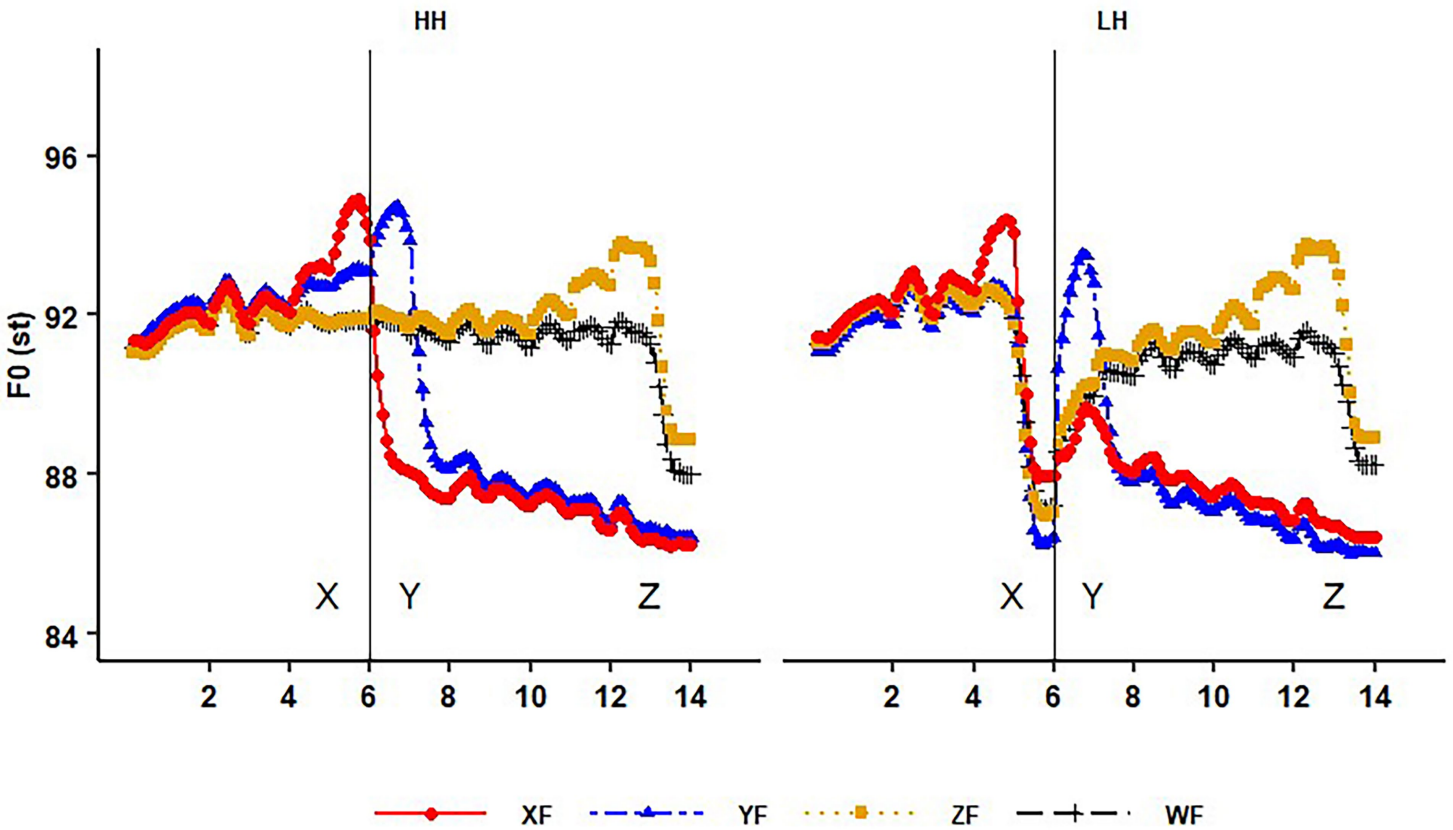
Time-normalized intonation contours of the HH (left) and LH (right) sentences in the conditions of the *syllable* boundary (between syllable X and Y), with the four focus conditions overlaid in one figure. Here XF, YF, ZF and WF stand for focus in word X, Y, Z and the wide focus condition. The *x*-axis are the syllable numbers. The vertical line indicates the critical boundary between X and Y.

**Figure 3:**
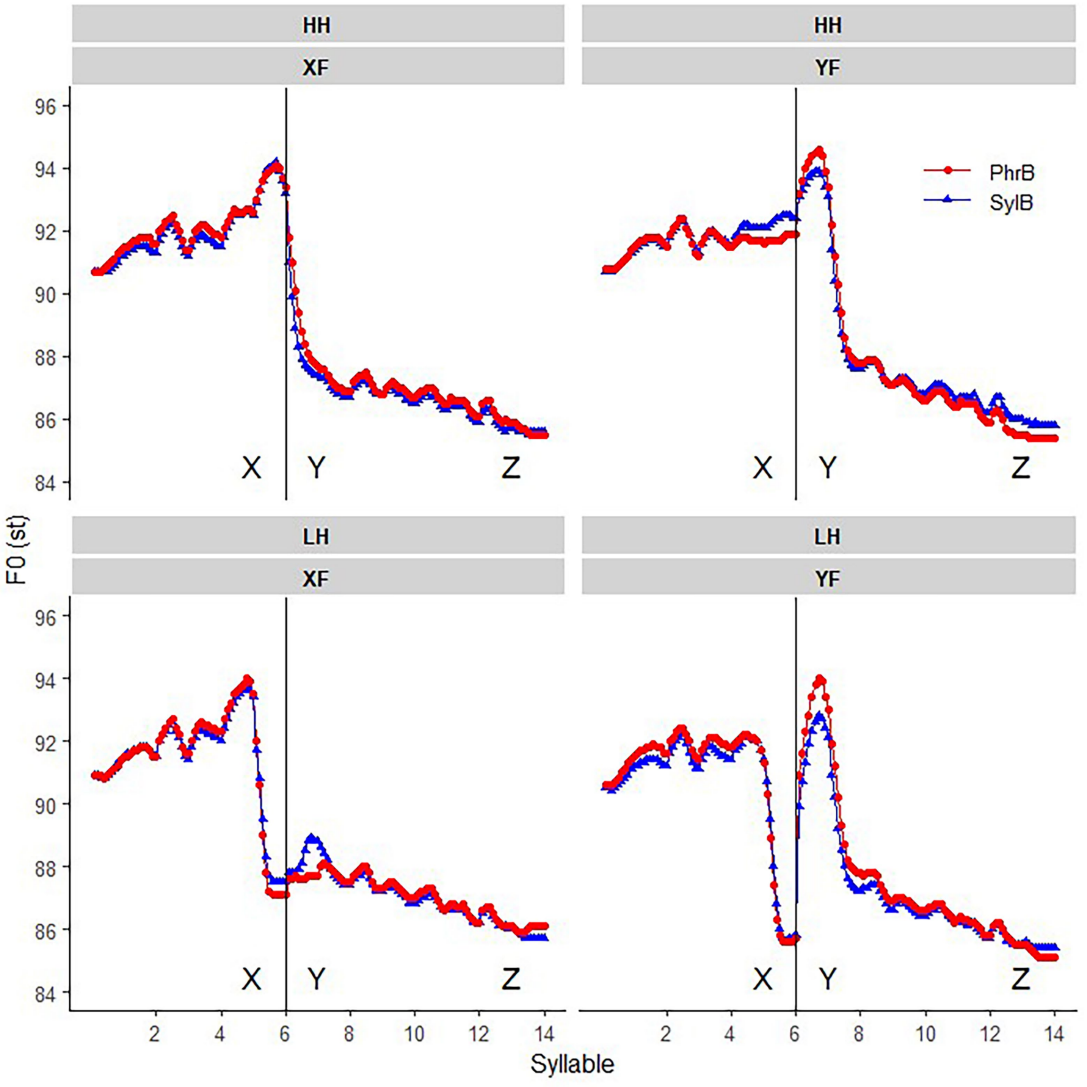
The time-normalized intonation contours of the two boundary conditions in the HH and the LH sentences under the XF and YF conditions. Here SylB and PhrB stand for syllable and phrase boundary between syllable X and Y. The *x*-axis are the syllable numbers.

**Figure 4:**
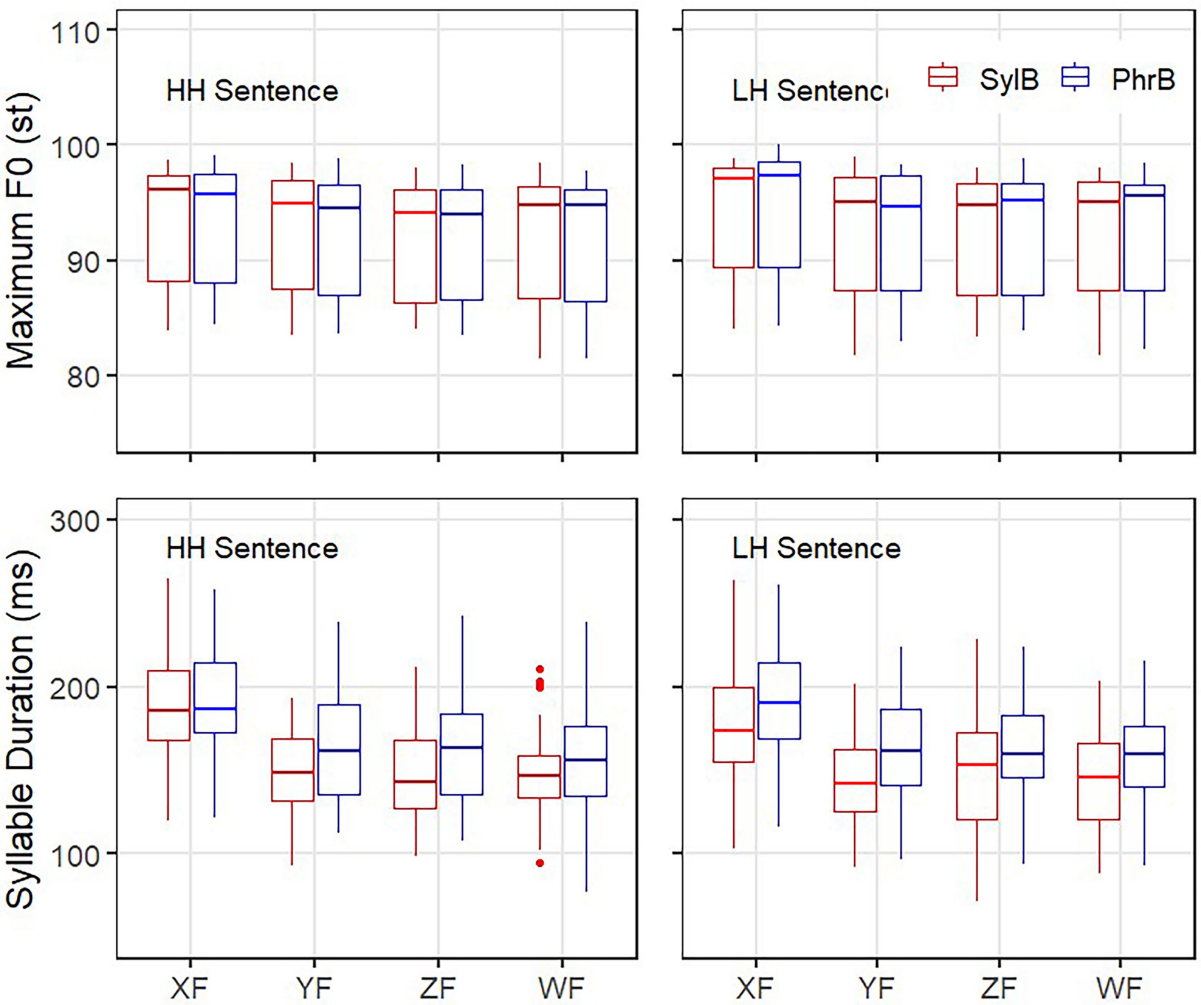
Maximum F0 and duration of syllable X in the H_X_H_Y_(left) and L_X_H_Y_(right) sentences in the two boundary conditions (SylB and PhrB) and the four focus conditions.

**Figure 5:**
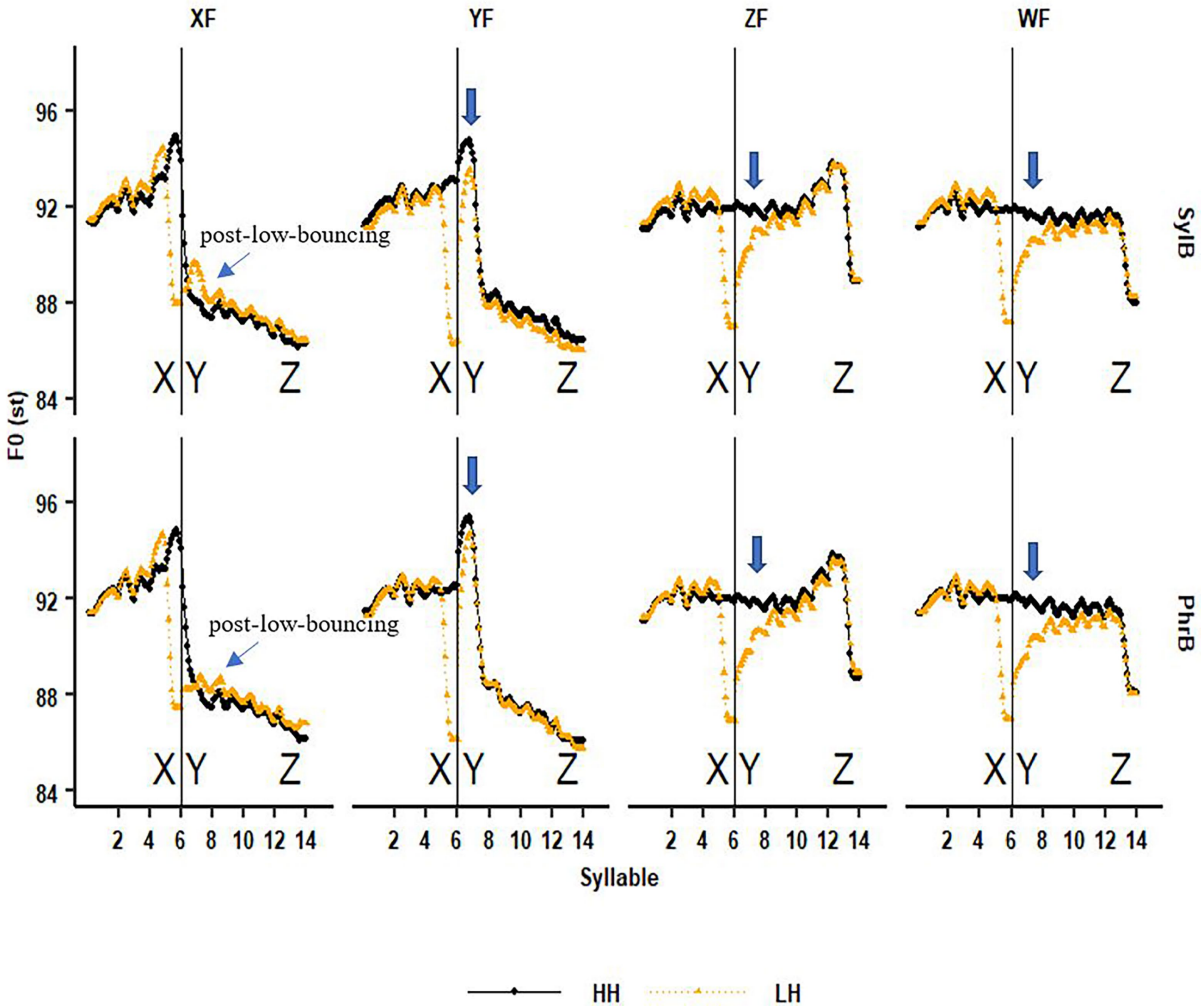
The comparison between the HH (black line) and LH (yellow line) sentences in four focus conditions (from left to right are the conditions of focus in syllable X, Y, Z and in wide-focus) under the condition that the boundary between X and Y is a syllable (SylB; upper row) or a phrase boundary (PhrB; lower row), as indicated by the vertical line. The downward arrow indicates where the downstep effect can be seen.

**Figure 6:**
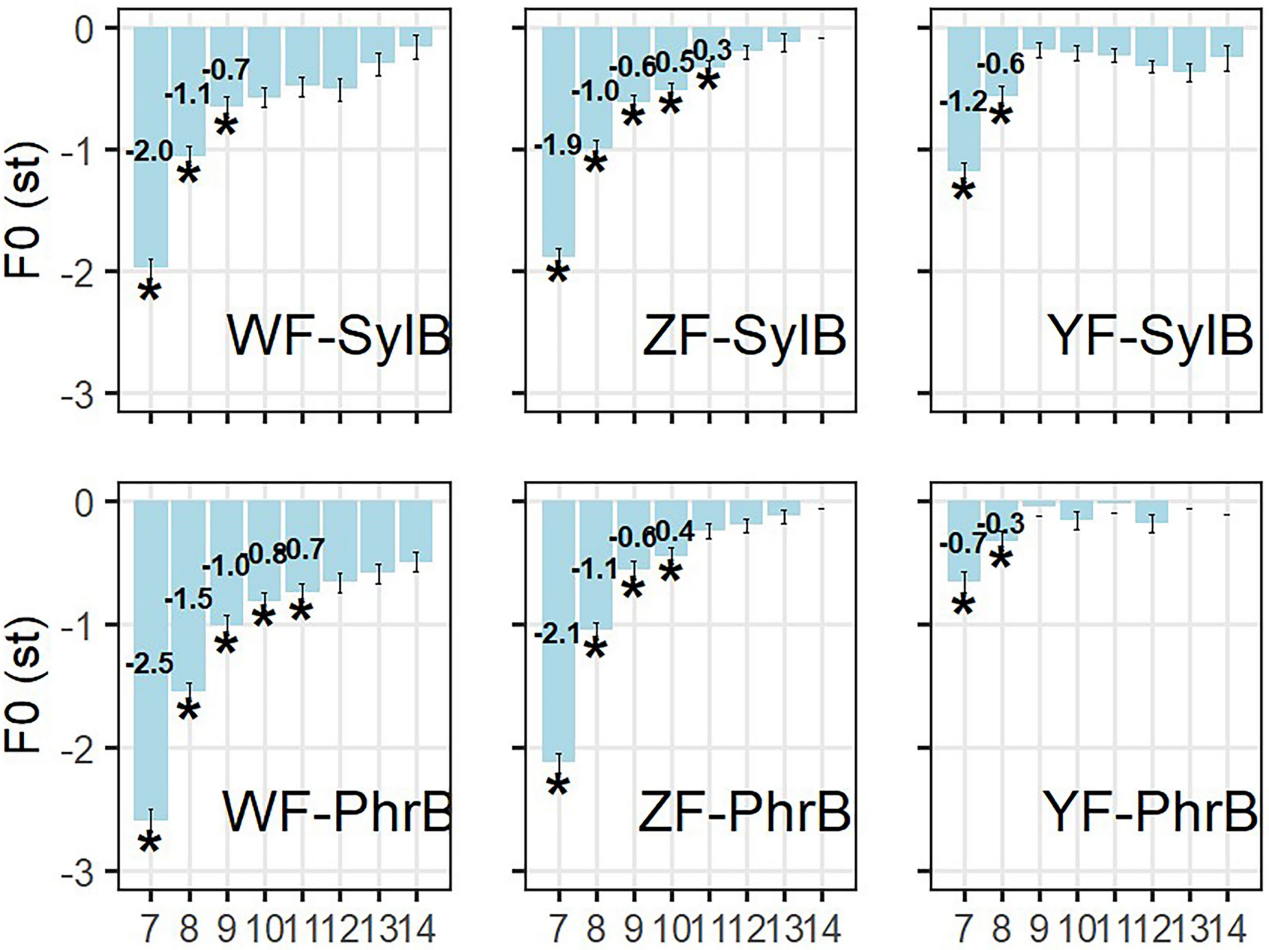
The downstep size in the Wide-focus (WF), Z-focus(ZF), and Y-focus (YF) conditions, divided by syllable (SylB) and phrase boundary (PhrB) conditions. The significant downstep effects are marked with ^*^ indicating that *p* < 0.05. The *x*-axis shows syllable numbers, in which the 7th is syllable Y, the H tone right after the L tone.

**Figure 7:**
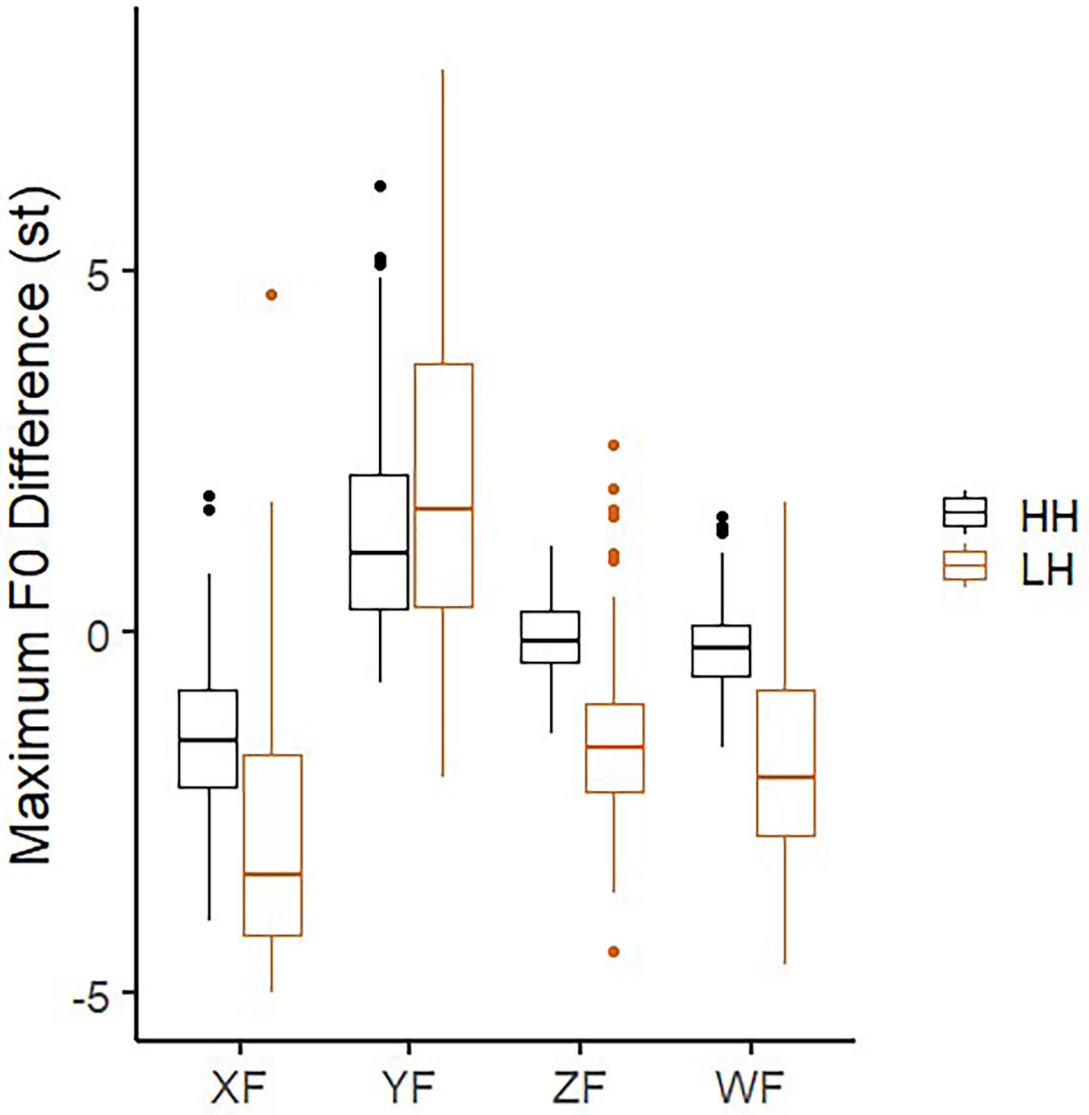
Maximum F0 difference between the two H tones surrounding syllable X (either L or H) in different focus conditions, while the two boundary conditions are averaged.

In this section, the graphic analysis firstly shows how focus and phrasing are realized in intonation (Figures 2, 3), followed by quantitative analysis of F0 and duration (Figure 4 and Table 1). These two sections serve to confirm that our results are largely consistent with previous studies on focus and phrasing, so that we are confident to further analyze their interaction with the tonal manipulation on intonation. To get an overview of the results, downstep and post-low-bouncing are firstly visually analyzed with intonation contours (Figure 5). Downstep is then quantitatively analyzed with two different methods, i.e., (a) the *cross- comparison* between LH and HH sentences to verify how many syllables it takes for the H tones after the L tone reaching the all-H sentences to answer Q1-Q4 (Figure 6 and Table 2); (b) the *sequential-comparison* between the H tones surrounding the L tone. By comparing the decrease of the H tones in the LH and HH sentence, we can tear apart the declination and the downstep effect to answer Q5 (Figures 7 8 and Table 3). Thirdly, we noticed that L tones are mostly creaky, especially in the phrase-boundary condition. Therefore, we aim at answering the question whether the change of phonation type to creaky voice is a cause on downstep. We then analyzed the pitch height in the H tone after the L tone as compared between the creaky and normal L tones to answer Q6 (Figure 9). We can show that the change of phonation type is not the direct cause of downstep. For post-low-bouncing, it only happens when the L tone is focused (XF). In line with the findings in Prom-on et al. (2012), we here provide further analysis on its interaction with boundary strength to answer Q7 (Figure 10). With this analysis, we can justify that the *balance-perturbation hypothesis* holds, which predicts weaker post-low-bouncing when the L tone is longer.

### Graphic analysis on focus and phrasing

First, we present intonation contours to show how focus is encoded in intonation. Figure 2 presents the HH and LH sentences in the condition of syllable boundary, with the 4 focus conditions overlaid in one figure. In each sentence, 10 time-normalized F0 points for each syllable were averaged across 48 observations (8 speakers × 2 sets × 3 repetitions).

We can see in Figure 2 that focus is realized as the tri-zone pattern as defined in Xu (1999) and repetitively found in many other studies (e.g., Wang et al., 2018b). Looking at the HH tone sentences, we can clearly see that the on-focus syllables show raised F0 and expanded pitch range; the post-focus words exhibit lowered and compressed pitch; while the pre-focus words are similar to the wide focus condition. It holds in the LH sentences as well, except that when the L tone word (e.g., ying1ou3) is focused (XF), the pre-low H is raised. And in the YF condition, on-focus F0 raising still applies in the H tone after the L tone. Thus, downstep does not override (or cancel) on-focus F0 raising. The sentences in the phrase boundary show a very similar pattern, which is not presented here for the interest of space. A phrase boundary does not block post-focus F0 compression (PFC), as likewise reported in Wang et al. (2018b). In general, it confirms that tonal interactions and phrasing do not change how focus is realized, though the amount of focal raising appears to differ between tone conditions.

Secondly, Figure 3 presents how boundary strength is encoded in intonation in the XF and YF conditions. No clear difference in F0 between the two boundary conditions can be seen here, in both the HH and LH (lower row) sentences. In WF and ZF conditions, the two boundary conditions do not show clear difference either, which is not presented here for the interest of space. It is in consistence with Wang et al. (2018b) that F0 plays a limited role on phrasing, especially on boundaries within a sentence. Importantly, when pre- and post-boundary syllables are under focus (the X and Y focus condition), there is still no clear sign of using F0 to mark boundary strength. Thus, focus in Mandarin does not seem to invulnerably insert a prosodic boundary.

The above graphic observations show that F0 variation is mainly triggered by focus and tone, but not by prosodic boundaries. We further analyzed the nature of the boundary and whether speakers distinguished the two boundary conditions phonetically. The following analysis of syllable duration (see section “Acoustic analysis on the interaction of focus and boundary,” Figure 4) confirms that boundary strength was encoded mainly in pre-boundary lengthening, but not F0.

### Acoustic analysis on the interaction of focus and boundary

From the graphic analysis (see Figures 2, 3), we can see that the intonation patterns of focus and phrasing are consistent with previous studies, e.g., Xu (1999) and Wang et al. (2018b). Since focus and boundary effects have already been extensively studied, statistical analysis on all the syllables is not presented here. Statistic test on syllable X is of particular interest as it interacts with downstep and post-low-bouncing. To better understand the interaction between boundary and focus on syllable X, we present the boxplot of maximum F0 and duration of syllable X in Figure 4.

Linear-mixed-models on maximum F0 and duration in syllable X were carried out in HH and LH sentences separately, with focus and boundary as two non-interactive fixed factors, while speaker and sentence set as random factors (see Table 1). Wide-focus and syllable-boundary were set as the baseline conditions. The LMM model was chosen to meet the criteria that (1) the model with presumed interaction did not show significant interactions, thus we took this model without interaction; and (2) it was with the lowest AIC and BIC while we tried different ways of setting the random effects.

**Table 1:** LMM analysis on maximum F0 and duration in syllable X, with HH and LH sentences separately tested taking focus and boundary as non-interactive fixed factors, whereas speaker and set as random factors in the equation as lmer(dv ~ focus+boundary+ (1|speaker) + (1|repetition) + (1|set), data = DT), here dv stands for dependent variable, which is MaxF0 and duration.

			HH				LH		
Random effects:		Num of Observations 429							
MaxF0		Var	SD			Var	SD		
	Speaker	24.89	4.98			29.15	5.40		
	Rep	0.06	0.25			0.04	0.21		
	Set	0.01	0.13			0.24	0.49		
	Res	1.10	1.05			1.22	1.10		
Duration									
	Speaker	165.57	12.87			181.46	13.47		
	Rep	2.14	1.46			6.07	2.46		
	Set	553.76	23.53			387.53	19.93		
	Res	1049.2	32.39			1305.3	36.12		
Fixed effects:									
		Est	SE	*df*	*t*	Est	SE	*df*	*t*
MaxF0	Inter	92.0	1.67	8.22	54.91^*^	91.57	1.84	8.63	49.75
	XF	2.80	0.14	413	19.58^*^	2.05	0.15	413	13.54^*^
	YF	1.44	0.14	413	10.08^*^	0.35	0.15	413	2.29^*^
	ZF	0.10	0.14	413	0.70	0.07	0.15	413	0.45
	PhrB	0.01	0.10	413	0.01	0.08	0.11	413	0.75
Dur	Inter	165.92	17.55	1.22	9.46^*^	168.55	15.30	1.36	10.97
	XF	66.08	4.41	416	14.98^*^	61.93	4.92	416	12.60^*^
	YF	24.41	4.41	416	5.53^*^	30.09	4.92	416	6.12^*^
	ZF	−7.19	4.41	416	−1.63	6.62	4.92	416	1.35
	PhrB	20.06	3.12	416	6.44^*^	23.58	3.48	416	6.78^*^

Note: * stands for *p* < 0.05.

As for focus effect on syllable X, together with the observations in Figure 4, the statistical analysis in Table 1 shows that focus significantly increases both maximum F0 (about 2.8 st) and duration (about 66 ms) of syllable X (see the line of XF in Table 1). In the Y focus condition, the 3 syllables HXY (H means the high tone before syllable X, e.g., ying1ou1dou1 ‘Yingou bag’) is possibly grouped as one prosodic word, thus syllable X is also with increased maximum F0 (about 1.4 st) and duration (about 24 ms; see the line of YF) in Table 1, which is in consistent with the findings in Chen (2006) on the durational domain of focus.

As for boundary effect on syllable X, the data in Table 1 (also see Figures 3, 4) show that boundary does not have any effect in maximum F0 (92.7 st vs. 92.6 st), but only in duration of syllable X (192 ms vs. 212 ms). No interaction was found between focus and boundary in the duration of syllable X, meaning that the pre-boundary lengthening applies to roughly the same degree in all the focus conditions (see Figure 4), which is in consistent with Wang et al. (2018b). The above results hold for both HH and LH sentences. It leads us to conclude that focus and tone do not interfere with pre-boundary lengthening. Thus, durational adjustment due to focus, boundary and tone is also largely encoded in parallel. We can then further test whether the lengthened L tone decreases or increases the level of downstep and post-low-bouncing in the following sections.

From Figure 3, we can see that maximum F0 in the L tone is actually the end point of the preceding H tone, which does not show any difference between the two boundary conditions (see Table 1 and Figure 4). Does the minimum F0 in the L tone differ between the boundary conditions? With similar LMM tests in the LH and HH sentences separately, taken boundary and focus as two fixed factors with interaction, and speaker as the random factor, the minimum F0 of syllable X in the LH sentence showed no difference in the two boundary conditions either (86.4 st on average in both conditions) (Estimate = −0.369, SE = 0.426, *df* = 352, *t* = −0.866, *p* = 0.387). However, there was an interaction between focus and phrasing, i.e., when the L tone is focused (XF), the minimum F0 in the phrase boundary condition is significantly lower than in the syllable boundary condition (Estimate = −1.402, SE = 0.598, *df* = 352, *t* = −2.344, *p* = 0.0196). In the other three focus conditions, no difference in minimum F0 was found between the two boundary conditions.

When we labeled the speech data, we noticed that most of the L tones were creaky, that was 84.1% and 74.6% in the phrase and syllable boundary, conditions respectively. It is possible that creakiness is an additional feature of a stronger boundary, when minimum F0 cannot go any lower at a phrase boundary (Kuang, 2017).

To summarize, (1) focus is reliably realized in a tri-zone pattern, i.e., pre-focus F0 is largely intact, on-focus F0 is raised and post-focus F0 is lowered and compressed; in addition, focus increases duration of the focused syllable; (2) boundary strength has very little effect on maximum or minimum F0, but mainly realized by pre-boundary lengthening, which is independent from focus and tone; (3) The L tone is more likely to be creaky when it is before a phrase boundary than a syllable boundary.

### Graphic analysis on downstep and post-low-bouncing

The analysis on focus and boundary in section “Graphic analysis on focus and phrasing” and section “Acoustic analysis on the interaction of focus and boundary” shows that the current experiment is in agreement with previous findings on these two effects (Xu, 1999; Wang et al., 2018b). It validates the following analysis on the interaction of these two functional variations with the tonal effects, i.e., downstep and post-low-bouncing. As introduced in the beginning of the results section, we here firstly report the *cross-comparison* on assessing the downstep effect adopted from Xu (1999) and Shih (2000) among many others, by comparing crossly between the HH and LH sentences (see Figure 5).

In the wide- and Z focus sentences, we can see in Figure 5 that F0 raises greatly in syllable Y in the LH sentence, which is the procedure of target approximation from a low starting point to the H target. As expected, F0 in syllable Y does not reach the height as the HH tone sentences in several H tones after the L tone, showing a clear downstep effect. We can also see that the downstep effect becomes weaker when the H tones are in a longer distance from the L tone. Five new findings are as below.

1. The downstep effect also holds when the focused word is sentence final (ZF), indicating that on-focus F0 raising in word Z does not seem to have any anticipatory effect on downstep.
2. The above observations hold in both the syllable and phrase boundary conditions. Thus, a stronger phrase boundary does not block the downstep effect. Despite a longer duration in the L tone before a phrase boundary (see Figure 4), downstep still applies. The following analysis shows that this is because the L tone is with lower F0 and even becomes creaky at a phrase boundary.
3. When the H tone right after the L tone is focused (YF), the downstep effect still shows in syllable Y but not in the following H tones. Surprisingly, even on-focus F0 raising does not cancel the downstep effect. In other words, we can say that downstep does not cancel on-focus F0 raising. It further confirms that the downstep effect is relatively robust. However, post-focus-compression (PFC) seems to override the downstep effect since there is no clear difference in the H tones after syllable Y between the HH and LH sentences, which is statistically confirmed below in Figure 6.
4. Comparing the two boundary conditions it seems that downstep is greater in the phrase boundary condition, however, in the YF condition the downstep effect is weaker in the phrase boundary condition.
5. When the L tone is under focus (XF), instead of downstep, the post-low-bouncing effect shows in the adjacent H tones. Here, the H tone after the L tone goes up first, then drops gradually, as compared to the all-H sequence, as reported in Prom-on et al. (2012). Note that the H tones in the baseline condition are realized lower, i.e., in a compressed pitch register (post-focal compression, Prom-on et al., 2012; Xu et al., 2012). The new finding is that the post-low-bouncing effect seems to be weaker in the phrase boundary condition than in the syllable boundary condition, which supports the *balance-perturbation hypothesis* as proposed in Prom-on et al. (2012).

In summary, the graphic analysis of Figure 5 shows that: (1) The downstep effect is relatively robust in varied focus and boundary conditions. More specifically, downstep is not blocked by a phrase boundary, neither is it overridden by on-focus F0 raising or phrase boundary. (2) When the L tone is under focus, post-low bouncing is found in the following H tones, and seems to be weakened by a phrase boundary.

### The *cross-comparison* of downstep effect

The main questions to be quantitatively analyzed are the size and the domain of downstep and post-low-bouncing effect, and their interactions with focus and phrase boundary.

Downstep is firstly analyzed by comparing the LH and the corresponding HH sentences in the WF, ZF and YF conditions. In the *cross-comparison,* the size of the downstep effect is calculated by the difference in maximum F0 between the H tones in the LH and HH sentence in syllable Y (syllable 7) and the following syllables (syllable 8 to 14). The post-low-bouncing effect is calculated in the X-focus condition in a similar way (see section “F0 analysis on post-low-bouncing effect”).

Figure 6 presents the size of downstep effect in the three focus and two boundary conditions. The mean values show how much F0 maximum is lowered in the LH sentence as compared to the HH sentence in the corresponding syllable. Paired-sample T tests were applied in each syllable to test whether the difference reached statistical significance at the level of *p* < 0.05, which is marked by a * in Figure 6.

To get an overall statistical analysis of the factors on the downstep effect, a LMM was applied, setting focus, boundary, and syllable as fixed factors with interactions presumed (WF, syllable boundary, and the 7^th^ syllable are set as the base-line condition), while speaker is the random factor (see Table 2). Putting it together with the t-test in Figure 6, the following findings are statistically supported: (1) The downstep effect in Y-focus condition is significantly smaller than that in wide-focus condition, while no difference is found between Z-focus and wide-focus condition. (2) The downstep effect decreases as the H tones are in longer distance from the L tone. (3) Unexpectedly, the downstep effect is greater in the phrase boundary condition than the syllable boundary condition, especially in the wide-focus conditions. It is probably because the L tone is with lower minimum F0 and with more creaky voice (see section “Acoustic analysis on the interaction of focus and boundary”), thus the following H tone is with a larger difference from the all-H reference, as compared to the syllable boundary condition. (4) In the Y-focus condition, the downstep effect interacts with focus and boundary, in the way that the downstep effect in the adjacent syllable of the L tone is greater in the syllable boundary condition than in the phrase boundary condition.

**Table 2:** LMM analysis on downstep size (difference of maximum F0 between LH and HH sentences in the H tones) with the equation as lmer(downstepsize ~ focus * syllable * boundary + (1 | speaker), data = DT2).

	Number of observations: 2544			
Random effects:		Variance	SD	
Speaker	(Intercept)	0.078	0.279	
Residual		1.218	1.104	
Fixed effects:	Estimate	SE	*df*	*t*
(Intercept)	2.84	0.27	435	10.47^*^
YF	−1.53	0.36	2,522	−4.30^*^
ZF	0.11	0.36	2,522	0.30
syllable	−0.20	0.02	2,522	−8.57^*^
PhrB	0.79	0.36	2,522	2.21^*^
YF:syllable	0.12	0.03	2,522	3.56^*^
ZF:syllable	−0.02	0.03	2,522	−0.69
YF:PhrB	−1.16	0.50	2,522	−2.31^*^
ZF:PhrB	−0.55	0.50	2,522	−1.08
syllable:PhrB	−0.04	0.03	2,522	−1.28
YF:syllable:PhrB	0.06	0.05	2,522	1.18
ZF:syllable:PhrB	0.02	0.05	2,522	0.45

Note: * stands for *p* < 0.05.

### The *sequential-comparison* on downstep and declination

Another way to analyze downstep is the degree of F0 lowering after a L tone. In this way, downstep effect can be compared with declination, which was analyzed by calculating the difference of the maximum F0 in the adjacent H tones in all-H sentences. Firstly, we just analyzed the two H tones surrounding the L tone, that is the difference of maximum F0 between syllable 7 and 5 (Difsy7sy5), presented in Figure 7 as boxplots divided by focus conditions, with HH and LH sentences compared directly. Since the results already show that boundary has no effect on maximum F0 in syllable X (Figure 4), we here averaged the two boundary conditions in Figure 7.

To evaluate whether there is declination in all H tone sentence, we compared “Difsy7sy5” in the wide focus condition of the HH sentences with 0 in a one-sample *t*-test (*t* = −3.359, *df* = 107, *p* = 0.001). The 95% confidence interval is −0.3 to −0.07. With the same analysis, however, declination is not found in the Z-focus condition (*t* = −1.46, *df* = 105, *n.s.*). Thus, declination is to a much less degree and vulnerable to be cancelled by a final focus.

The LMM model on “Difsy7sy5,” with focus, boundary and tone as fixed factors and speaker as random factor, showed a main effect in tone and focus, but not in boundary (see Table 3). It further confirms that F0 plays a limited role on differentiating boundary degrees.

**Table 3:** LMM analysis on the difference of maximum F0 in the H tones before and after syllable X, with focus, boundary and tone as fixed factors, whereas speaker as a random factor in the formula as: difs7s5 ~ tone * focus + boundary + (1 | speaker).

	Number of observations: 858			
Random effects:		Variance	SD	
Speaker	(Intercept)	0.3804	0.6167	
Residual		1.7171	1.3104	
Fixed effects:	Estimate	SE	*df*	*t*
(Intercept)	−0.138	0.245	14.755	−0.56
toneLH	−1.655	0.179	841.013	−9.214^*^
XF	−1.264	0.178	840.997	−7.088^*^
YF	1.724	0.178	841.000	9.649^*^
ZF	0.101	0.179	841.011	0.566
boundaryPhrB	−0.103	0.089	841.001	−1.149
toneLH:XF	−0.084	0.253	841.005	−0.332
toneLH:YF	2.326	0.253	841.013	9.181^*^
toneLH:ZF	0.298	0.253	841.009	1.178

Note: * stands for *p* < 0.05.

In general, by comparing HH with LH in Figure 7, we can see that the difference on the degree of F0 drop in the all-H tone sentence is significantly less than the downstep effect in XF, ZF and WF conditions (*p* < 0.05). The interaction between focus and tone is not found in XF condition. We can see that Difsy7sy5 is greater in LH than in the HH sentence. Besides, Difsy7sy5 in the HH sentence is much smaller in the XF than in the WF condition, which reflects post-focus-compression (PFC) in F0. The new finding here is that downstep still shows aside from PFC. It means that the downstep effect is not just the general downtrend of F0. Declination and downstep are presumably not from the same articulatory mechanism.

When focus is on the H tone after the L tone (YF), the pitch difference between the two H tones (syl5 and syl7) is greater in the LH than the HH sentences. This comes from pre-low-raising (Lee et al., 2021). Here, it also shows the pre-low-raising is independent of on-focus F0 raising.

**Figure 8:**
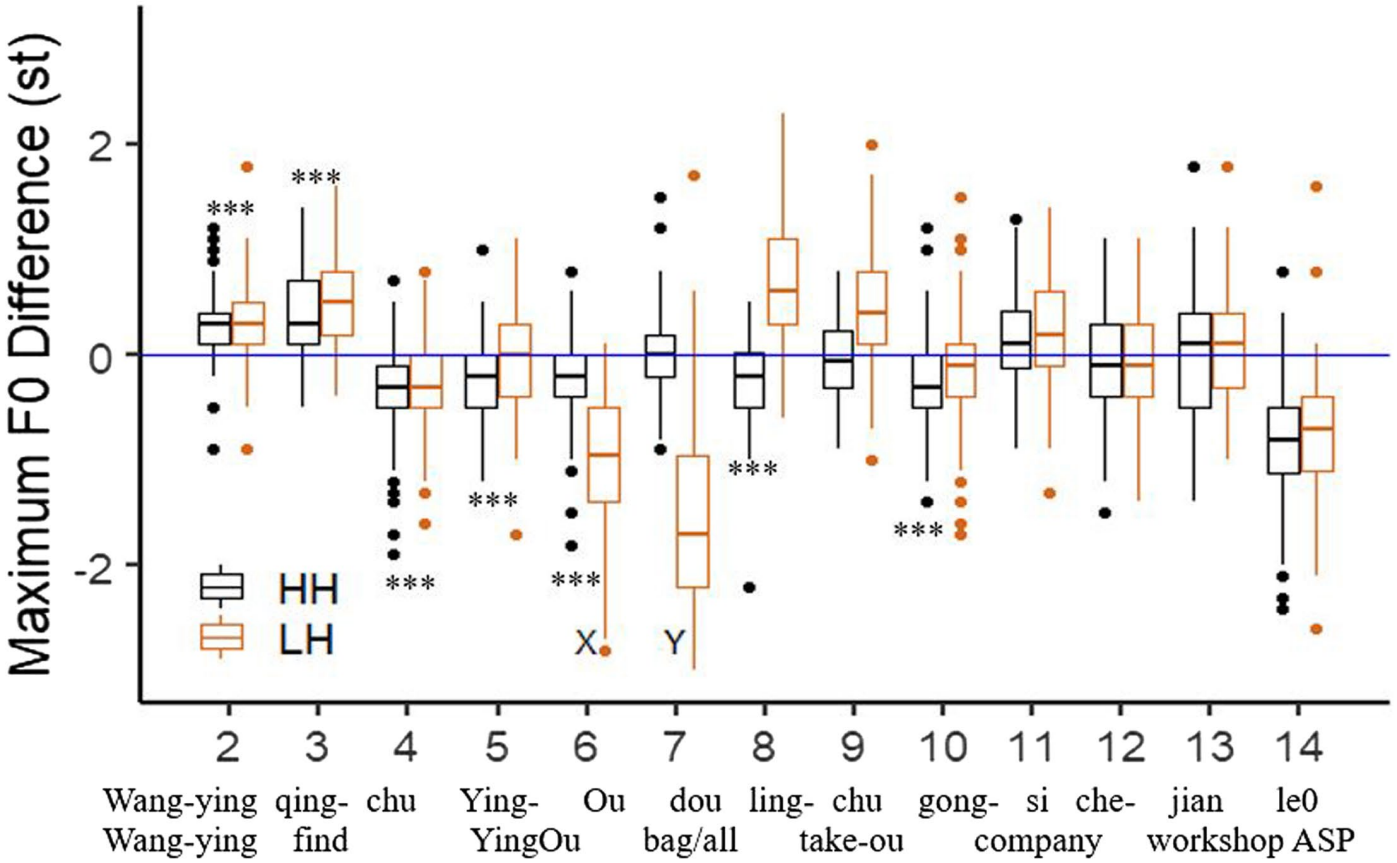
Boxplot of the maximum F0 difference between two adjacent H tones, as compared between HH and LH wide-focus sentences. The number in the *x*-axis (2–14) means that this is the maximum F0 of the current syllable minus the preceding syllable. ^***^ here indicates significant difference between 0 by one-sample *t*-test in the HH sentences.

Then, we further tested whether declination holds all along the sentence by comparing maximum F0 of each adjacent H tones, see Figure 8. We here only consider the wide-focus condition. The *** in the figure indicates that the F0 raise or drop between two adjacent H tones in all-H sentence is greater than 0 by on-sample *t*-test with *p* < 0.001, otherwise there is no difference between the two H tones. To put it in a simple way, the *** means that there is either F0 raising or declination in the current syllable. We can see that in the HH sentences, F0 goes up in the beginning of the sentence (increased 0.34 st), then drops gradually for about 3 syllables (decreased 0.25 st). However, between syllable 7 and 6 and between syllable 9 and 8, no significant difference is found in maximum F0. These two positions are phrase boundaries. It is possible that a phrase boundary cancels declination. Toward the end of the sentence, declination is absent as well. The last syllable is a neutral tone, which causes a sharp drop in F0. Thus, declination is with a very small pitch drop between two H tones, and can be easily cancelled due to topic, boundary, tone and other reasons.

If we look at the LH sentences, we can see that the H tones around the L tone causes much greater F0 change than the all-H sentence. Toward the end of the sentence, the adjacent H tones do not differ in F0, which is similar to the all-H sentence. It is in agreement with the *cross-comparison* of the downstep effect, that downstep gets weaker as the H tones are further from the L tone.

All-together, both the *cross-* and *sequential-comparison* show that downstep effect is in a much greater degree than declination. Downstep effect is robust, lasting for about 2–3 syllables. Downstep effect is not cancelled by focus or boundary, whereas declination can be cancelled by these two informative functions.

### Creaky L tone

**Figure 9:**
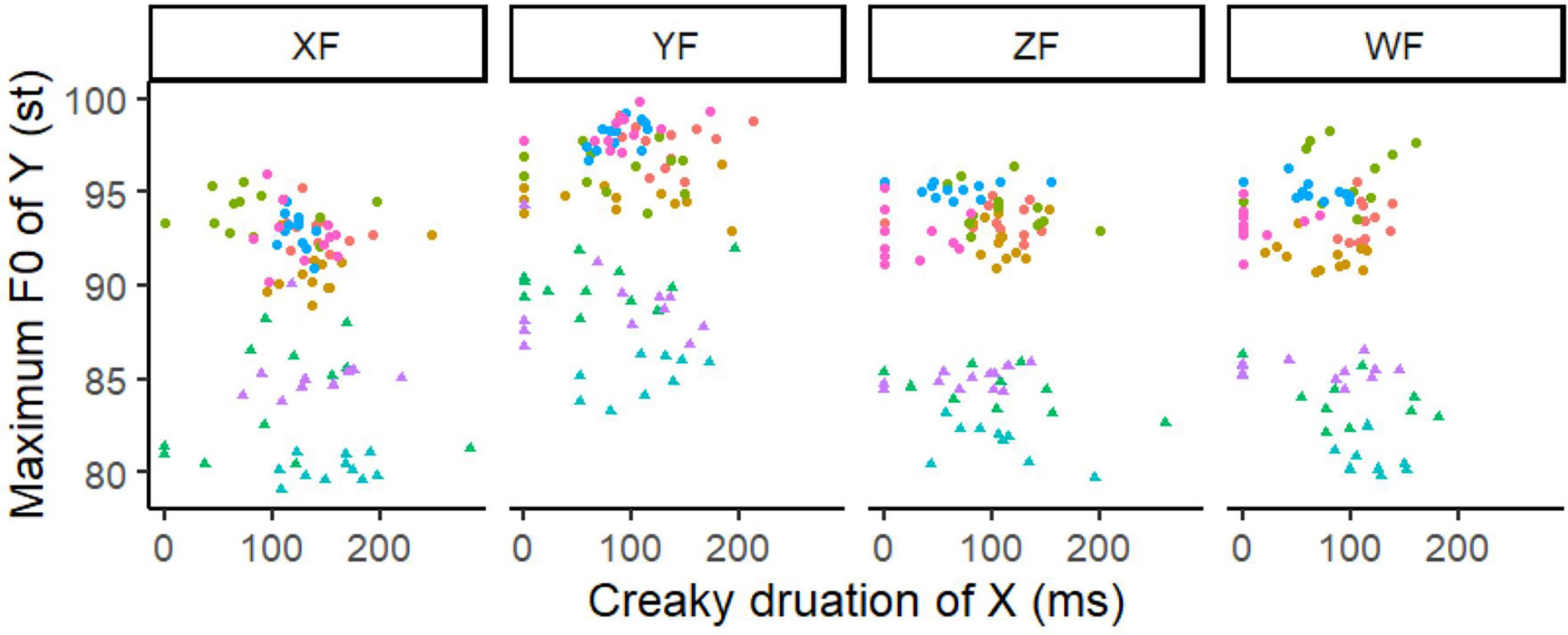
Scatter plot of the maximum F0 in syllable Y as functioned by duration of the creaky part in syllable X, with the color and shape differentiating speakers. The focus conditions are divided in each plot. The points with creaky duration of X being 0 means that this is a normal L tone.

The very last question concerning downstep is whether it is caused by creaky voice, or whether creaky L tones cause greater downstep effect. The number of creaky L tone in different conditions is presented in Table 4. In line with previous studies, we also see that when the L tone is under focus and at a phrase boundary, it is more likely to be creaky. We do not go into detailed analysis on the acoustic parameters of the creaky L tone. Instead, we simply calculate the amount of creakiness in L tones to answer the question whether a creaky low tone causes greater downstep effect. In Figure 9, the maximum F0 of syllable Y is plotted against the duration of the creaky part in syllable X, with four focus conditions divided in different plots. When the creaky duration is 0, it means this is a normal L tone. Here we do not see any clear trend of a creaky L tone causes lower F0 in the following H tone, which is supported by the LMM model analysis with creaky, focus and gender as fixed factors and speaker as a random factor (lmer(maxF0syl7 ~ Creakylablel*focus*Gender+ (1|speaker), data = creaky)). The LMM shows significant effect in focus and gender, whereas creaky does not show any effect (Estimate = −0.2455, SE = 0.416, *df* = 347, *t* = −0.59, *n.s.*). Thus, creaky L tone is not the direct cause of downstep, but strengthens downstep. It then explains why downstep effect is greater after a phrase boundary (Figure 6).

**Table 4:** The percentage of creaky L tone in different focus and boundary conditions (%).

	Syllable boundary	Phrase boundary
XF	95.8	91.6
YF	60.4	72.9
ZF	70.8	85.4
WF	64.5	83.3

### F0 analysis on post-low-bouncing effect

**Figure 10:**
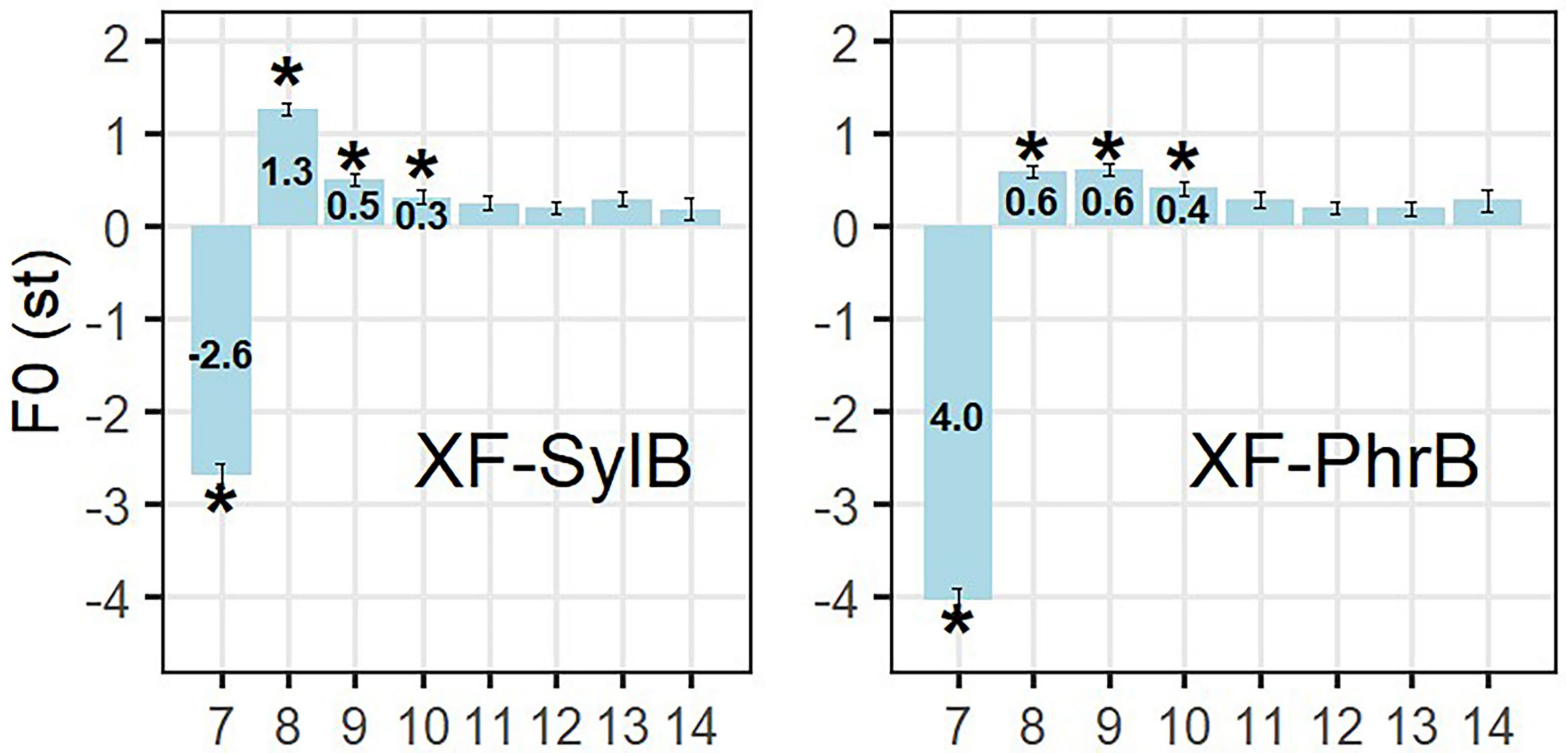
Post-low-bouncing effect in the X-focus condition when the boundary between syllable X and Y is either a syllable (SylB) or a phrase (PhrB) boundary. The *x*-axis shows syllable numbers, in which the 7th is syllable Y, the H tone right after the L tone.

The post-low-bouncing effect was calculated as the difference of maximum F0 in the H tones between the LH and HH sentences in the XF condition (the L tone is on-focus). As can be seen in Figures 7, 10, F0 maximum is lower in the syllable right after the L tone (syllable 7), that is because the F0 maximum of syllable 7 in the HH sentence is the offset of the previous H tone (the maximum F0 hence appears at the onset of the syllable 7 representing the transition from the focused H tone to a post-focally H tone). Post-low-bouncing shows at the end of syllable 7, which can be observed in the maximum F0 of syllable 8 and 9, then the pitch gradually drops back. The linear-mixed-model analysis was carried out with syllable and boundary as two fixed factors (with interaction), while speaker and set are random factors. In this statistical test, we only considered syllable 8 to 10, since no difference is shown between HH and LH sentences after the 10^th^ syllable (see Figure 10). The LMM analysis shows a significant effect in syllable (SE = 0.093, *t* = −5.087*), boundary (SE = 1.193, *t* = −3.018*) and the interaction (SE = 0.132, *t* = 2.899*). Thus, the following observations in Figures 5, 10 are statistically supported: (1) The post-low-bouncing effect gradually decreases in the syllables after the L tone; (2) A phrase boundary weakens post-low-bouncing effect, especially in the second H tone after the L tone.

## General discussion

The new contribution of the current study is on how pragmatic functions interacts with downstep and post-low-bouncing. With the control of focus, post-low-bouncing was brought in, which was mainly analyzed for neutral tones in previous studies (Chen and Xu, 2006; Prom-on et al., 2012). In the current study, it happened in the following H tones when the L tone is focused (XF in Figure 5). Although, a large part of intonation variation is informative, we want to emphasize that articulatory constrains on pitch change could not be neglected, since post-low-bouncing and downstep last for several syllables with decreasing in size from of 2.5 to 0.5 st, interacting actively with phrasing and focus. Our findings support the *additive division hypothesis* of pitch range, proposed in Liu et al. (2021). They found that pitch range of 5–12 st above the baseline signals both focus and surprise, suggesting an overlap between different layers of meanings within this pitch range. In their study, to perceive focus, F0 needs to be raised about 3 st. We here show the downstep and post-low-bouncing are in a pitch range of less than 2.5 st (Figures 6, 10), whereas on-focus F0 raising in syllable X is 2.8 st on average (Table 1). In a rough sense, it explains why on-focus F0 raising needs to be about 3st, beneath which F0 variation reflects tone and articulatory constrains. It is possible that any sudden and great pitch raising may bring-in informative meaning, thus it takes several syllables for downstep and post-low-bouncing to go back to the reference line. The process of target approximation as proposed in PENTA model (Xu et al., 2022) probably reflects both articulatory and perceptual constrains.

Relating to the tonal variation due to the L tone, the pre-low-raising was systematically studied in Lee et al. (2021) in Thai and Cantonese, that is, the H tone is raised in pitch before a L tone. They discussed three possible explanations: (a) a velocity account, (b) a perceptual account, and (c) an anatomical account. More specifically, (a) the raising pitch in the preceding syllable may increase the distance of the downward movement toward the low tone; (b) pre-low-bouncing may enhance tonal contrasts to aid comprehension; (c) if pre-low-raising is not actively planned, it may be the direct result of intrinsic laryngeal muscle movement. Their analysis does not support (b), the perceptual account. Putting it together with Prom-on et al. (2012) and the current study, we can conclude that pitch movements caused by a L tone (pre-L-raising, downstep and post-low-bouncing) are largely the outcome of intrinsic and extrinsic laryngeal muscle movement. Below we will provide detailed discussion on the research questions.

### How do focus and boundary interact with downstep (Q1-Q6)?

This is actually a very complicated question, since focus in Mandarin involves both on-focus raising and post-focus-compression in F0 (Shih, 1988; Xu, 1999; Chen and Gussenhoven, 2008; Wang and Xu, 2011; Wang et al., 2018b), see Figure 2 in the current study. Besides, downstep refers to the relevant pitch height in the H tones, either as compared to all-H reference line (*cross-comparison* answering Q1-Q4), or as the F0 drop between the H tones before and after the L tone (s*equential-comparison* answering Q5). To make the question even more complicated, L tones usually become creaky (Q6). No previous study has considered the influence of creakiness on downstep (Q6). Thus, the first question is split to the following 6 sub-questions, aiming to fully understand the property of downstep, and to take apart declination and downstep. The results are interpretable and coherent to each other if we take the idea that downstep is mostly constrained by articulatory movement, instead of conveying linguistic meaning.

#### Q1: Does downstep set up a new register tone?

The answer is No. The original motivation of this study was whether downstep in Mandarin can be modelled as a phonetic or as a phonological tonal interaction. On the one hand, downstep was observed in West-African tone languages (Welmers, 1959). The downstepped H tone defines a new ceiling for subsequent tones which was interpreted as a systematic, phonological effect, and downstep was phonologically modelled in terms of register tones (Snider, 1998) or register features (Akumbu, 2019). On the other hand, if downstep were a phonetic effect, the expectation is that the locally lowered F0 raises gradually back to its original register line. The present study suggests that downstep in Mandarin is indeed a phonetic tonal interaction. We observed that after a L tone, F0 does not raise back to the height of the all-H-tone sentences and lasts for several H tones decreasing in size, as has been repeatedly found in previous studies in Mandarin (Shih, 1988; Xu, 1999). The locally induced tonal interaction smoothly levels out such that the original reference line for a high tone in Mandarin is reached again (Figure 6). Thus, downstep in Mandarin is different from those in African languages. Moreover, our data showed that the effect size and the domain of the effect vary as a function of focus and prosodic boundary in Mandarin.

#### Q2: Does a sentence-final focus ends downstep?

We predicted that the answer is no because downstep is presumably local, and pitch target of each tone is realized syllable-by-syllable as stated in PENTA model (Xu et al., 2022). Indeed, we found that a late focus does not end downstep. Unexpectedly, downstep effect lasts longer in the Z-focus condition than in the wide focus condition (Figure 6). This is probably different from Hausa (Lindau, 1986), in which downstep can be canceled in yes/no questions. It is possible that speakers try not to cause confusion, otherwise any pitch raising before the final word may increase the prominence level in that word, given that sentence-final focus is quite similar to wide focus intonation (Xu, 1999; Liu and Xu 2007; Xu et al., 2012). Since the study on Hausa concerns question intonation, whereas ours is on final-focus, a controlled study of downstep in yes-no-questions in Mandarin would shed more light on this case.

#### Q3: Is downstep eliminated by on-focus F0 raising and post-focus-compression?

We predicted that informative functions of intonation may override an articulatory effect. However, the results show that downstep is only weakened by on-focus F0 raising and post-focus-compression but not fully cancelled. This result is new. It indicates that downstep, as an articulatory pitch movement, is pretty robust. According to Xu and Sun (2002), the time of pitch rise can be estimated by *t* = 89.6 + 8.7 *d* (here *d* stands for the change of pitch in semitone). Using this algorithm, we calculated the estimated time from the minimum F0 of the L tone to the maximum F0 of the following H tone. The exact duration of the H tone is actually longer than the estimated time (mean = 33 ms, sd = 35.6). It means that the observed downstep is not because of time pressure. When the H tone is focused (YF), the exact H tone duration is 60 ms (sd = 47.8) longer than the estimated time, however downstep effect still shows (Figures 5, 6). It further confirms that even in the condition of a longer H tone, downstep still applies. Thus, we draw the conclusion that informative intonation functions do not override downstep. The interaction between focus and downstep is gradual.

#### Q4: How does a phonological phrase boundary interact with downstep?

As predicted, the pre-boundary L is lengthened at a phrase boundary (Figure 4), the tonal target is fully realized with higher frequency of being creaky (Table 4), and in turn, it leads to greater downstep effect (see Figure 6). In wide focus condition, the L tone is lengthened about 14 ms in the phrase boundary, with no difference in minimum F0 between the two boundary conditions (86.7 vs. 86.1 st). Instead, creaky L tone occurs more frequently in the phrase boundary condition than the syllable condition (84% vs. 67%). That might be the reason why the H tone is a little lower in the phrase boundary condition than in the syllable boundary condition (90.1 st vs. 90.4 st), showing as a greater downstep effect under the phrase-boundary condition. However, creakiness *per se* does not seem to cause downstep (see below, Q6).

#### Q5: Do declination and downstep share the same mechanism?

The answer to this question actually depends on how to measure declination and downstep. It also remains controversial whether there is any separate articulatory mechanism of declination. We here take the *sequential-comparison* by calculating the difference of adjacent H tones (Figures 7, 8). As predicted, we can see that downstep and declination come from different articulatory control. However, it is not because downstep is local whereas declination is global, rather downstep lasts for several syllables as well. It is because the downstep effect shows in a larger scale and in a more robust manner than declination. It is possible that there is some underlying articulatory control on declination, however, it is pretty weak and vulnerable to be overridden by varied reasons. We are in agreement with other studies (Xu, 1999; Shih, 2000; Yuan and Liberman, 2014), showing that the general global downtrend, as modelled with a top and bottom regression line of intonation, is a combined effect from different functions. We further suggest not to just take the global downtrend in an abstract way, but to analyze it with full consideration of local tonal interactions.

#### Q6: Is creaky voice the cause of downstep?

Downstep is caused by a L tone, which is usally creaky in Mandarin (Kuang, 2017). Is it possible that creaky voice is the main cause of downstep? In our study we found that the L tone is more likely to be creaky when it is under focus and before a phrase boundary (Table 4). It confirms the claim by Kuang (2017) that creaky voice correlates with low pitch target. As discussed in Q4, more creaky L tones at a phrase boundary causes greater downstep effect. However, normal L tone causes roughly the same degree of downstep, as showed in the LMM that creakiness does not have any effect on the maximum F0 of the following H tone. No correlation is found between the duration of the creaky part in L tones and the pitch height in the following H tones (Figure 9). It indicates that creaky voice is probably not the direct cause of downstep. A normal L tone also causes downstep. However, a creaky L tone leads to a greater downstep effect.

### Does a phrase boundary block post-low-bouncing (Q7)?

According to the *balance-perturbation hypothesis* (Prom-on et al., 2012), we predicted that post-low-bouncing is weakened if the L tone is at a phrase boundary. It is indeed the case, as shown in Figure 7. They hypothesized that after producing a very low F0, the extrinsic laryngeal muscles (e.g., sternohyoids) stop contracting to maintain the balance between the two antagonistic forces in the intrinsic laryngeal muscles. When the L tone is focused, the extra force may cause a sudden increase of the vocal fold tension, resulting in the raise in F0 in the following H tone. We here see that when the L tone is before a prosodic phrase boundary, it then probably gives a little more time to release the tension between the extrinsic and intrinsic laryngeal muscles. This would explain the difference in size of post-low bouncing found in our data. In line with Prom-on et al. (2012), we also found that post-low-bouncing occurs in H tones when the L tone is under focus. In their study, neutral tones after a L tone show post-low-bouncing. The reason might lie in the fact that post-focal words are weakened in intensity and compressed in F0. The weakened H tones at post-focal position might share some similar mechanism with weak articulatory movement in the neutral tones.

At last, we here briefly introduce some preliminary findings in the current study, relating to Moisik et al. (2014). They have found that low F0 tone targets in Mandarin can not only be reached by lowering the larynx, but also by combining the raise of larynx height and laryngeal constriction, which may lead to creakiness in the low tone. In the L tone, the amount of F0 lowering correlates with larynx lowering in male speakers (r = 0.73 and 0.86), while the female speaker uses larynx raising (*r* = 0.13; Figures 11-13, pp. 39 in their study). In our study, the minimum F0 of the low tone (X) is positively correlated to the maximum F0 of the following H tone (Y) in the male speakers (wide focus: *y* = −2 + 0.99*x*, *r*^2^ = 0.66; X-focus condition: *y* = 9.4 + 0.87*x*, r^2^ = 0.736), but not in the female speakers (wide focus: *y* = 65 + 0.27*x*, r^2^ = 0.073; X-focus condition: *y* = 79 + 0.12*x*, r^2^ = 0.01). To fully understand the anatomical process in downstep, articulatory studies considering gender difference are required.

## Conclusion

To answer all the research questions concerning the interaction of focus/boundary with downstep/post-low-bouncing, we can draw the following conclusions.

In the wide focus condition, the downstep effect lasted for 3 syllables and gradually reached back to the all-H tone reference line. Downstep thus does not set up a new reference line in Mandarin (Q1). A sentence-final focus makes the downstep effect last for 5 syllables (Q2). When the H tone right after the low tone was focused (YF), on-focus F0 raising and post-focus-compression (PFC) weakened downstep (Q3). A phrase boundary strengthened downstep (Q4). We further analyzed downstep by measuring the F0 drop between the two H tones surrounding the L tone (*sequential-comparison*). Comparing it with F0 drop in all-H sentences, it showed that the downstep effect was much greater and more robust than declination (Q5). However, creaky voice in the L tone was not the direct cause of downstep (Q6). At last, when the L tone was under focus (XF), it caused a post-low-bouncing effect on the following H tones and lasted for about 3 syllables with F0 dropping back gradually. Moreover, post-low-bouncing is weakened by a phonological phrase boundary (Q7).

In general, this study showed that downstep and post-low-bouncing, as articulatory controls and local tonal interaction effects, interact with the execution of sentence-level pragmatic functions like focus and prosodic boundary. Pragmatic effects do not cancel or override articulatory effects, but affect the size and domain of the tonal interactions.

## Data availability statement

The raw data supporting the conclusions of this article will be made available by the authors, without undue reservation.

## Ethics statement

Ethical review and approval was not required for the study on human participants in accordance with the local legislation and institutional requirements. The patients/participants provided their written informed consent to participate in this study.

## Author contributions

FK initiated the general research question. BW, FK, and SG designed the experiment together. BW checked the labeling of the wav files, wrote the paper, and finalized the data analysis, closely working together with FK. SG did a preliminary graphic and statistical analysis. All authors contributed to the article and approved the submitted version.

## Funding

The experiment was supported by SFB632 “Information Structure” at Potsdam University. The writing procedure was further supported by Social Science Foundation of China to BW (18BYY079). Goethe University Frankfurt provided financial support for open access support, and a DFG grant KU 2323-4/1 to FK supported further assistance.

## Conflict of interest

SG is employed by i2x GmbH.

The remaining authors declare that the research was conducted in the absence of any commercial or financial relationships that could be construed as a potential conflict of interest. The research was initiated while SG was affiliated with Potsdam University, and hence the research was conducted without a potential conflict of interest.

## Publisher’s note

All claims expressed in this article are solely those of the authors and do not necessarily represent those of their affiliated organizations, or those of the publisher, the editors and the reviewers. Any product that may be evaluated in this article, or claim that may be made by its manufacturer, is not guaranteed or endorsed by the publisher.

## Supplementary material

The Supplementary material for this article can be found online at: https://www.frontiersin.org/articles/10.3389/fpsyg.2022.884102/full#supplementary-material
