# Supplementary material for: The interaction of focus and phrasing with downstep and post-low-bouncing in Mandarin Chinese
Source: Front Psychol. 2022 Sep 30;13:884102. doi: 10.3389/fpsyg.2022.884102 (PMC9561885; doi:10.3389/fpsyg.2022.884102)
Supplement: Supplementary file 1 [file Data_Sheet_1.docx]

# Appendix I

Reading Materials

| Focus questions | Target sentences |
| --- | --- |
| Set 1: Syllable boundary between syllable X(H/L) and Y(H) | |
| 你没听说吗？ (Wide Focus)  Have you heard about it? | 汪英清出(樱欧H兜H)拎出公司车间了。  汪英清出(樱藕L兜H)拎出公司车间了。  Wangying found an Yingou (H/L) bag and took it out of the workshop of the company. |
| 不是樱**安**兜吗？ (X-Focus)  Is it not Ying**an**dou? |  |
| 不是樱(欧H/藕L)**包**吗？ (Y-Focus)  Is it not an Yingou **sack**? |  |
| 不是**楼道**吗？ (Z-Focus)  Is it not corridor？ |  |
| Set 1: Phrase boundary between syllable X(H/L) and Y(H) | |
| 你没听说吗？ (Wide Focus)  Have you heard about it? | 汪英清出(樱欧H)都H拎出公司车间了。  汪英清出(樱藕L)都H拎出公司车间了。  Wangying found an Ying**ou (H/L)** and took all of it out of the workshop of the company. |
| 不是樱**安**兜吗？ (X-Focus)  Is it not Ying**an**dou? |  |
| 不是**部分**吗？ (Y-Focus)  Is it not **some** of it? |  |
| 不是**楼道**吗？ (Z-Focus)  Is it not corridor？ |  |
| Set 2: Syllable boundary between syllable X(H/L) and Y(H) | |
| 你没听说吗？ (Wide Focus)  Have you heard about it? | 安妮宣称(花微H莺L)该推出新包装了。  安妮宣称(花尾L莺H)该推出新包装了。  Anni claimed that Huawei(H/L) bird should launch a new package. |
| 不是花溪莺吗？ (X-Focus)  Is it not Hua**xi**ying? |  |
| 不是花微/尾雕吗？ (Y-Focus)  Is it not an Huawei (H/L) carving? |  |
| 不是工艺吗？ (Z-Focus)  Is it not technology？ |  |
| Set 2: Phrase boundary between syllable X(H/L) and Y(H) | |
| 你没听说吗？ (Wide Focus)  Have you heard about it? | 安妮宣称(花微H)应H该推出新包装了。  安妮宣称(花尾L)应H该推出新包装了。  Anni claimed that Huawei(H/L) should launch a new package.  Note: “should” in Chinese is either “该gai1” or “应该ying1gai1”. |
| 不是花溪莺吗？ (X-Focus)  Is it not Hua**xi**ying? |  |
| 不是不该吗？ (Y-Focus)  Is it that they should not? |  |
| 不是工艺吗？ (Z-Focus)  Is it not technology？ |  |
